# A MnO_2_-based tumor-seeking nanoplatform for enhanced chemoimmunotherapy against 4T1 breast cancer

**DOI:** 10.1016/j.mtbio.2025.102000

**Published:** 2025-06-17

**Authors:** Tingting Gong, Xiaohuan Wang, Ziqi Liu, Pengxin Li, Yunqian Lu, Yaoyao Guo, Meihua Han, Xiangtao Wang

**Affiliations:** a Institute of Medicinal Plant Development, Chinese Academy of Medical Sciences & Peking Union Medical College, No. 151, Malianwa North Road, Haidian District, Beijing 100193, China; b Research Center of Pharmaceutical Engineering Technology, Harbin University of Commerce, Harbin, Heilongjiang province, 150076, China; c School of Pharmacy, Henan University of Chinese Medicine, Henan province, 450046, China

**Keywords:** MnO_2_ nanoparticles, Immune remodeling, Anti-Tumor, Chemoimmunotherapy, Breast cancer

## Abstract

A significant obstacle in oncological therapy lies in surmounting the immunosuppressive microenvironment while enhancing the anti-tumor efficacy of chemotherapeutic agents. STING agonists such as Mn^2+^ have demonstrated substantial promise in this regard. Squamocin (Squ), a naturally derived compound, exhibits potent anti-tumor activity with minimal drug resistance; however, its application is hampered by poor water solubility (approximately 7.5 μg/mL) and off-target toxicity. In this study, a chemo-immunotherapy approach utilizing MnO_2_ nanoparticles combined with squamocin (Squ) has been formulated, with its therapeutic effectiveness further augmented through surface modification with the tumor-targeting IR820 molecule. The intravenously injectable MnO_2_ nanoparticles were synthesized from KMnO_4_ and anhydrous ethanol, and subsequently modified with Astragalus polysaccharides (APS)-IR820 on their surface to improve stability in physiological media and facilitate tumor targeting and *in vivo* fluorescence imaging. The resultant MnO_2_@APS-IR820 NPs, measuring 193.4 ± 1.7 nm, generated Mn^2+^ in response to the elevated glutathione (GSH) levels within the tumor microenvironment. The generated Mn^2+^ notably induced maturation of bone marrow dendritic cells (BMDCs) and promoted the repolarization of tumor-associated macrophages from the pro-tumor M2 phenotype to the anti-tumor M1 phenotype. When combined with Squ@APS-IR820 NPs (220.1 ± 11.2 nm in size), the chemoimmunotherapy significantly stimulated both innate and adaptive immune responses in a 4T1 tumor bearing mouse model and demonstrated a synergistic anti-tumor effect, achieving a tumor inhibition rate of approximately 92 %, highlighting its remarkable potential for anti-tumor therapy.

## Highlights

• Potential STING agonists MnO_2_ nanoparticles were prepared using KMnO_4_ and ethanol.
• Chemically synthesized tumor targeting fluorescent coating materials APS-IR820.
• APS-IR820 serves as a tumor-seeking encapsulating material to formulate nanoparticles.
• Chemoimmunotherapy combining Squ and MnO_2_ exhibits synergistic therapeutic efficacy.

## Graphical abstract

Amplifying STING activation with chemo-immunotherapy combining MnO_2_@APS-IR820 NPs and Squ@APS-IR820 NPs for cancer treatment. Created in BioRender. Gong, T. (2025) https://BioRender.com/itwim3r. MnO_2_@APS-IR820 NPs and Squ@APS-IR820 NPs accumulated in tumor tissue through the guide of IR820, where MnO_2_@APS-IR820 NPs degrades and produces Mn^2+^ in response to H^+^ and GSH in TME. The released Mn^2+^ was uptaken by cells through ion channel (such as DMT1), and acts as agonist of cGAS-STING signaling [[1], [2]]. In addition, Mn^2+^ can effectively activate cGAS-STING pathway, and subsequently activate transcription factors IRF3 to express IFN. This process initiates innate and adaptive anti-tumor immunity. Squ, squamocin; APS, astragalus polysaccharides; TME, tumor microenvironment; m DC, mature dendritic cells; im DC, immature dendritic cells; GSH, glutathione; GSSG, glutathione disulfide; IFN, interferon; TNF, tumor necrosis factor. NK cell, natural killer cell; MDSC, myeloid-derived suppressor cell; DMT1, divalent metal transporter 1; IRF3, interferon regulatory Factor *3*; NF-κB, nuclear factor kappa-B.

**Figure:**
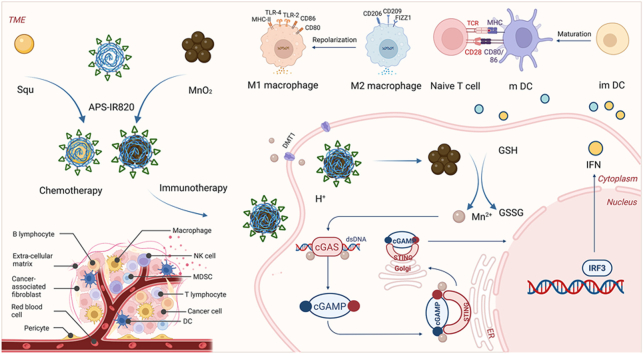

## 1 Introduction

The tumor microenvironment (TME) comprises tumor cells, the recruited immune cells and inflammatory cells, tumor associated fibroblasts, adjacent stromal tissue, microvessel, and a variety of cytokines and chemokines secreted by these cells [3], which interact with each other to maintain immunosuppressive microenvironment and foster tumor progression, such as inducing tumor proliferation, angiogenesis, and distant metastasis, and facilitating cancer cells evolution towards drug resistance and evasion of host immune surveillance [[4], [5], [6]], and ultimately undermining the therapeutic efficacy of chemotherapy, radiotherapy, and immunotherapy [[7], [8], [9], [10]]. Therefore, remoulding the tumor microenvironment and relieving immune suppression has become the basis of anti-tumor therapies [11].

The cGAS-STING pathway contributes to initiate anti-tumor immunity by inducing type I interferon production in response to exogenous dsDNA. The activation of this pathway can promote tumor specific antigen presentation, macrophage repolarization, and cytotoxic lymphocytes (CTL) activation, further enhancing the anti-tumor immune response [2,12]. STING agonists could be mainly divided into conventional cyclic dinucleotide (CDN)-based STING agonists, non-CDN small molecule STING agonists, and Mn^2+^. Traditional CDN STING agonists, due to their poor stability and poor permeability, can only be administered intratumorally [13,14], unsuitable for the treatment of non-solid tumors and metastatic tumors. Meanwhile, CDN synthesis is difficult, costly, and clinically ineffective [15]. Non-CDN small molecule agonists are also difficult to synthesize due to their complex structures [16].

Mn^2+^ was verified as an potential novel agonist of the cGAS STING pathway[[17], [18], [19]], which could compensate aforementioned shortcomings of CDN and non-CDN small molecule STING agonists. But Mn^2+^ lack tumor targetability and TME responsiveness, and fail to effectively remould TME. Some researchers have addressed these issues through nanotechnology. For instance, Zhou et al. [20] developed manganese-phenolic networks via the coordination of tannic acid with manganese ions, followed by surface modification with bovine serum albumin (BSA) to improve colloidal stability. This network structure not only acts as a drug delivery carrier but also achieves precise release of the drug and Mn^2+^ respond to pH/GSH of tumor cells. MnO_2_ nanoparticles are another kind of advantageous potential STING agonist, which have attracted much attention due to their ability to produce Mn^2+^ in response the high concentrations of GSH (Eq. (1)) and H^+^ in TME.

(1) $${MnO}_{2}+2H^{+}+2GSH={Mn}^{2+}+GSSG+2H_{2}O$$

The resultant Mn^2+^ can act as an agonist of the cGAS STING pathway [17,18], which could induce dendritic cell maturation, T cell infiltration, and neutrophil repolarization [21]. Furthermore, MnO_2_ NPs are simple to synthesize and adaptable to multiple administration routes for *in vivo* delivery, which enhances the synergistic anti-tumor treatment in combination with chemotherapy, radiotherapy, and so on. Bare MnO_2_ nanoparticles are prone to aggregate in physiological media such as 5 % glucose solution, saline, and PBS, highlighting the necessity for surface modification, such as polyacrylamide hydrochloride (PAH), polyacrylic acid (PAA) [22], PEG [23], polylysine (PLL) [9], etc.

Polysaccharides, bioactive macromolecules with good biocompatibility, biodegradability, biosafety, diverse pharmacological activities, and wide availability from natural sources, are potential biomaterials for the surface modification to MnO_2_ nanoparticles. Astragalus polysaccharides (APS) [24] are soluble polysaccharides extracted from the roots of *Astragalus membranaceus*, with a mean molecular weight of 150 kDa. In our preliminary study, the surface modification of APS effectively improved the stability of MnO_2_ nanoparticles. In order to confer tumor targeting and fluorescent imaging capability of MnO_2_ nanoparticles, APS was further conjugated with a tumor-seeking fluorescent molecule IR820, a heptamethine cyanine dye that shares similar structure and function with Cy7 dyes [25,26]. The meso-Cl group of IR820 can be displaced by a free thiol in albumin at the Cys34 site in blood, and the resultant albumin adducts accumulate in tumors via the EPR effect**.** Furthermore, organic anion transporting polypeptides (OATPs) contribute to the uptake of tumor-seeking dyes by cancer cells [27]. IR820 and its conjugates exhibit strong tumor targeting and long-term tumor retention properties [27,28].

Squamocin (Squ, Fig. S1) is a natural, potent anti-tumor agent isolated from *Annona Squamosa* seeds, which regulates histone H3 phosphorylation levels by modulating aurora B and pERK in cancer cells to induce G1 phase arrest and apoptosis in cancer cells [29]. Squamocin (Squ) inhibits tumor cell growth at nanomolar IC50 values, demonstrating 10–100-fold higher potency compared to first-line antitumor drugs such as paclitaxel [30]. However, poor solubility [31] severely limits its applications. Fortunately, with the assistance of oleic acid (OA), Squ could be successfully encapsuled by APS and APS-IR820 into nanoparticles for *in vivo* drug delivery.

Here, MnO_2_ nanoparticles were prepared via a novel one-step method using KMnO_4_ and anhydrous ethanol and further surface-modified with APS-IR820 to achieve tumor-targeting capability, fluorescence imaging, and TME responsiveness. Meanwhile, Squ was encapsulated into nanoparticles by APS-IR820, which in combination with MnO_2_@APS-IR820 nanoparticles demonstrated excellent synergistic anti-tumor efficacy.

## 2 Reagents and methods

### 2.1 Reagents

Potassium permanganate (KMnO_4_) was purchased from Beijing Yili Fine Chemicals Co., Ltd (Beijing, China). Anhydrous ethanol and anhydrous methanol were obtained from Tianjin Beilian Fine Chemicals Co., Ltd (Beijing, China). IR820-dihexanoic acid was obtained from Tianjin Heowns Technology Co., Ltd (Tianjin, China), IR820 hexanoic acid was simplified as IR820 in the article. Oleic acid (OA) was provided by Beijing Puyihua Technology Co., Ltd (Beijing, China). Astragalus polysaccharides (APS) was obtained from Tianjin Seno Pharmaceutical Co., Ltd (Tianjin, China). NaBH_3_CN was bought from Shanghai Macklin Biochemical Technology Co., Ltd (Shanghai, China). EDCI was obtained from Shanghai Haohong Biomedical Technology Co., Ltd (Shanghai, China). Ethylenediamine (EDA) and DMAP was bought from Shanghai Aladdin Biochemical Technology Co., Ltd (Shanghai, China). Anhydrous Dimethyl sulfoxide (DMSO) was purchased from J&K Technology Co., Ltd (Beijing, China). Squamocin (Squ) was extracted from the seeds of *Annoma squamosa* and purified in our lab [32]. RPMI 1640 medium, Phosphate buffer saline (PBS), fetal bovine serum (FBS), Penicillin streptomycin, Collagenase IV, and trypsin was bought from Thermo Fisher Scientific Co., Ltd (Massachusetts, USA). S 3-(4,5-dimethylthiazol-2-yl)-2,5-diphenyl-2H-tetrazolium bromide (MTT) and GSH assay kit and lipopolysaccharide (LPS) was obtained from Beijing Solarbio Science & Technology Co., Ltd (Beijing, China). Fixation buffer and permeablization buffer were provided by Beijing Lanjieke Technology Co., Ltd (Beijing, China). Hyaluronidase, DNAse Ⅰ, and Percoll were purchased from Shanghai Macklin Biochemical Technology Co., Ltd (Shanghai, China). Zombie Aqua™ Dye was obtained from Biolegend (CA, USA). Recombinant Mouse IL-4 Protein, Recombinant Mouse CSF-2/GM-CSF Protein, and Recombinant Mouse CSF-1/M-CSF Protein was provided by Abclonal (Wuhan, China). CF488 Tunel Cell Apoptosis Detection Kit was provided by Servicebio Technology Co., Ltd (Wuhan, China).

Antibody: Anti-mouse CD16/32 Antibody was provided by Elabscience Biotechnology Co., Ltd, Anti-mouse CD11c PE, anti-mouse CD80 FITC, anti-mouse CD86 APC, anti-mouse CD86-PE, anti-mouse CD45-APC was purchased from Proteintech Group, Inc (Hubei, China). Cell staining buffer was obtained from Shanghai Maokang Biotechnology Co., Ltd. Anti-mouse CD 45 APC-cy7, anti-mouse CD3 Bv421, and anti-mouse Gr1(Ly6G + Ly6c) PerCP-Cy 5.5 were provided by Becton, Dickinson and Company (New Jersey, USA). Anti-mouse CD4 FITC, anti-mouse CD8 Bv786, anti-mouse B220 FITC, anti-mouse CD11c PE-cy7, anti-mouse CD80 PE, anti-mouse CD86 PE-CF594, anti-mouse CD206 FITC, anti-mouse Ly6c PE-CF594 were obtained from Biolegend (CA, USA). Anti-mouse F4/80 PerCP-Cy5.5 was purchased from Elabscience (Wuhan, China).

### 2.2 Cell lines and animals

The 4T1 (murine mammary carcinoma) cell line was obtained from the China Infrastructure of Cell Line Resource (Beijing, China). 4T1 cells were cultured in RPMI 1640 medium supplemented with 10 % FBS at 37 °C in a 5 % CO_2_ atmosphere. Penicillin-streptomycin (1 % v/v) was added to the medium. Female Balb/c mice aged 6–8 weeks (22 ± 2 g) were provided by SiPeiFu Biotechnology Co., Ltd. (Beijing, China). All animal experiments were conducted in compliance with principles of the Ethical and Regulatory Guidelines for Animal Experiments established by IMPLAD, Beijing, China. The study was approved by the Ethics Committee of IMPLAD. The animals were raised in an SPF facility (Temperature: 25 ± 3 °C, Humidity: 70 % ± 5 %, 12 h light/12 h dark cycle) for one week prior to experimentation.

### 2.3 Synthesis and characterization of MnO_2_ nanoparticles (MnO_2_ NPs)

MnO_2_ NPs were synthesized via oxidation-reduction reaction. Specifically, 50 mg of potassium permanganate (KMnO_4_) was dissolved in deionized water and reacted with anhydrous ethanol at ambient temperature. After continuous stirring for 12 h, nanoparticles were isolated via centrifugation at 8000 rpm for 10 min. Subsequent purification involved multiple washing cycles, with repeated dispersion in deionized water and centrifugation until the supernatant became completely clear, ultimately yielding purified MnO_2_ nanoparticles.

The infrared spectra of MnO_2_ NPs were obtained using an FT-IR spectrometer (Thermo Nicolet 6700, USA). 5 mg of lyophilized MnO_2_ NPs was mixed with potassium bromide (KBr), and the mixture was ground until homogeneous. The homogeneous mixture was then placed into a pellet die and subjected to sufficient pressure to obtain a thin pellet suitable for direct analysis.

X-ray photoelectron spectroscopy (XPS) analysis was performed using an Energy Dispersive X-ray Spectroscopy (Thermo Fisher, ESCALAB 250Xi, USA) with a 100 eV (Step size: 1 eV) and 20 eV (Step size: 0.1 eV) pass energy for the survey and high-resolution scans to identify the chemical composition of MnO_2_ NPs. The XPS spectra of MnO_2_ NPs was processed by smoothing, baseline correction, and peak fitting for spectral characterization.

### 2.4 Synthesis and characterization of APS-IR820

#### 2.4.1 Synthesis of APS-IR820

##### 2.4.1.1 Preparation of ammoniated APS

Ammoniated *Astragalus Polysaccharides* (APS-EDA) were synthesized by dissolving 100 mg APS, 100 mg EDA and 40 mg NaBH_3_CN in PBS (pH 8.0) with stirring at room temperature for 72 h. Then anhydrous ethanol was added until the ethanol concentration reached 80 %, followed by centrifugation at 6000 rpm for 20 min. The sediment was collected, redispersed into deionized water, and precipitated in 80 % ethanol, followed by centrifugation to collect the precipitate. This washing procedure was repeated four times. Finally, the resulting sediment was dissolved in deionized water and dialyzed against 50 % ethanol for 48 h using a dialysis membrane with a molecular weight cutoff (MWCO) of 8000–14000 Da, with the external dialysate replaced every 12 h. Then the aqueous solution in dialysis bag was freeze-dried to harvest ammoniated APS.

##### 2.4.1.2 Preparation of APS-IR820

5 mg IR820-dihexanoic acid, 10 mg EDCI, and 2 mg DMAP were dissolved in 0.5 mL of anhydrous DMSO and magnetically stirred for 1 h to activate the carboxyl groups of IR820-dihexanoic acid. Then 50 mg of ammoniated APS was dissolved in 5 mL anhydrous DMSO and added to the aforementioned mixture. The reaction was allowed to proceed in the dark under a nitrogen atmosphere for 48 h. Finally, anhydrous ethanol was added to the reaction system to achieve an 80 % ethanol concentration. The mixture was centrifuged at 6000 rpm for 20 min to collect the crude APS-IR820, which was then subjected to a resuspension-centrifugation procedure four times.The resulting product was dissolved in deionized water and dialyzed against 50 % ethanol for 48 h (MWCO: 8000–14000 Da) before being freeze-dried to obtain APS-IR820.

IR820 was dissolved in a mixed solution of DMSO and deionized (DI) water (3:7, v/v) and then serially diluted to prepare a series of working solutions (1, 1.25, 2, 2.5, 5, 7.5, 10 μg/mL) for fluorescence intensity measurement (λ_ex_ = 710 nm, λ_em_ = 833 nm) and calibration curve plotting. APS-IR820 was dissolved in the same DMSO-water mixture at a concentration of 1 mg/mL for fluorescence intensity measurement. Based on the calibration curve of IR820, the IR820 content in APS-IR820 was determined.

#### 2.4.2 Characterization of APS-IR820

##### 2.4.2.1 Fourier transform infrared spectroscopy (FT-IR)

The FT-IR spectra of APS, APS-EDA, IR820, and APS-IR820 were obtained using an FT-IR spectrometer (Thermo Nicolet 6700, USA) scanning from 4000 cm^−1^ to 400 cm^−1^ with a resolution of 4 cm^−1^. Thirty-two scans were averaged for each spectrum. The KBr slice of APS, APS-EDA, IR820, and APS-IR820 was prepared following the same method as that used for MnO_2_ NPs as in section 2.3.

##### 2.4.2.2 Visible (Vis) spectrum

The Vis spectra of APS, APS-EDA, IR820, and APS-IR820 were recorded on a Cary UV–Vis spectrophotometer (Varian/Agilent Technologies, Switzerland) from 900 to 400 nm at 1 nm interval, using the corresponding solvent as a control. For the measurements, APS, APS-EDA, and APS-IR820 were dispersed in DI water, while IR820 was dissolved in methanol.

##### 2.4.2.3 Fluorescence properties

DMSO and DI water was mixed with a volume ratio of 3:7, aiming to dissolve all the samples in the same solvent. APS, APS-EDA, IR820, and APS-IR820 were dissolved in the mixed solvent (DMSO: DI water = 3:7 (v/v)). Emission spectra was obtained from 750 nm to 900 nm using a FL970 spectrofluorometer (Techcomp Scientific Instruments Co., Ltd, China, λ_ex_ = 710 nm).

### 2.5 Preparation of nanoparticles

#### 2.5.1 Preparation of MnO_2_@APS and MnO_2_@APS-IR820

7.5 mg MnO_2_ NPs were dispersed in DI water at a concentration of 1.5 mg/mL, dropped slowly in 5 mL APS aqueous solution (7.5 mg/mL) under ultrasonication (250 W, Kunshan Kemeng Ultrasonic Technology Co., Ltd, Kunshan, PR China), followed by centrifugation at 8000 rpm for 10 min to collect MnO_2_@APS NPs. MnO_2_@APS-IR820 NPs were prepared according to the same procedure except for the replacement of APS with APS-IR820.

#### 2.5.2 Fabrication of Squ@-APS and Squ@APS-IR820

3 mg Squ and 15 mg OA were dissolved in 4.5 mL methanol. Then, 450 μL of the resulting methanol solution was added dropwise to 2 mL APS solution (6 mg/mL) under ultrasonication. Methanol was subsequently removed by evaporation under low pressure (40 mbar) at 40 °C, yielding Squ@APS NPs (Squ: OA: APS = 0.2:1:8, mass ratio). Squ@APS-IR820 NPs were prepared according to the same procedure except for the replacement of APS with APS-IR820. When Coumarin 6 (C6) was incorporated into the methanol solution of Squ, the same procedure obtained Squ/C6@APS NPs and Squ/C6@APS-IR820 NPs (Squ:OA:APS or APS-IR820:C6 = 0.2:1:8:0.03, mass ratio) for the subsequent cellular uptake study.

### 2.6 Characterization of nanoparticles

The particles size, PDI value, and Zeta potential of MnO_2_ NPs, MnO_2_@APS NPs, and MnO_2_@APS-IR820 NPs, Squ@APS NPs, and Squ@ APS-IR820 NPs were measured using a Malvern Instruments (Zetasizer Nano ZS, UK) at 25 °C.

10 μL of diluted nanoparticles were deposited onto a 230-mesh copper network, and allowed to settle for 5 min. The samples were snapped by a transmission electron microscope (Ruli TEM HT7800, Tokyo, Japan) at an accelerating voltage of 80 kV after being negatively stained by 0.5 % (w/v) phosphotungstic acid. The distribution of Mn and O was visualized by scanning TEM (STEM, FEI Tecnai G2 F30) and the corresponding energy-dispersive X-ray spectroscopy (EDS) mapping was analyzed on an Energy Dispersive Spectrometer (Oxford Xplore 30).

2 mL of Squ@APS and Squ@APS-IR820 NPs was lyophilized and weighed, and the lyophilized powder was dissolved in 18 mL chromatographic methanol and centrifuged at 12,000 rpm for 10 min to completely release Squ from the nanoparticles. The supernatant was analyzed by HPLC to determine the total Squ content in lyophilized powder. Nanoparticles (0.4 mL) were put into an Ultra centrifugal filter (0.5 mL, MWCO 10 kDa, Millipore, USA) and centrifuged at 12,000 rpm for 10 min. The filtrate was analyzed by HPLC for free Squ content. The drug loading capacity (DLC) and encapsulation efficiency (EE) was quantified by a high-performance liquid chromatography detector (UltiMate 3000, DIONEX, USA) at 208 nm using a UV–VIS spectrophotometer. Chromatographic condition consisted of Venusil XBP C18 (L) column (4.6 mm × 250 mm, 5 μm), mixture of 0.3 % phosphoric acid and acetonitrile as a mobile phase.

Drug loading capacity (DLC) (Eq. (2)) and encapsulation efficiency (EE) (Eq. (3)) was calculated according to the following equations:

(2) $$DLC\%=W_{1}/W\times 100\%$$

(3) $$EE\%=\left(W_{1}-W_{f}\right)/W_{1}\times 100\%$$

Where W1 and W are the weight of Squ encapsuled in the NPs and the weight of the lyophilized NPs. Wf are the weight of Squ in the filtrate.

### 2.7 Stability

#### 2.7.1 Storage stability

The Squ@APS, Squ@APS-IR820, MnO_2_@APS, and MnO_2_@APS-IR820 nanoparticles were stored at room temperature. Particle size and PDI were monitored at 0, 1, 2, 4, 6, 8, 10, 12 h. Meanwhile, turbidity, aggregation or precipitation of nanoparticles was recorded.

#### 2.7.2 Stability in physiological media

The Squ@APS, Squ@APS-IR820, MnO_2_@APS, and MnO_2_@APS-IR820 nanoparticles were mixed with 1.8 % NaCl, 10 % Glu, and 2 × PBS at a 1:1 vol ratio, followed by incubation in a 37 °C water bath. Then the mixtures were sampled at 0, 1, 2, 4, 6, 8, 10, and 12 h to measure their particles size and PDI.

### 2.8 pH/GSH-dependent decomposition of MnO_2_ nanoparticles

MnO_2_ is stable under neutral and weakly alkaline conditions but readily decomposes into Mn^2+^ in acidic conditions. Meanwhile, manganese dioxide exhibits oxidizing properties, enabling it to react with GSH to generate Mn^2+^, especially under weakly acidic conditions, as illustrated in Eq. (1).

The UV–VIS absorbance spectrum of MnO_2_@APS NPs was scanned from 200 nm to 800 nm and the degradation of MnO_2_ could be determined by the decrease of MnO_2_-characteristic absorbance at pH 7.4 and pH 5.5, which simulate normal tissue pH and weakly acidic pH in TME. In brief, MnO_2_@APS NPs were placed under different pH and incubated at 37 °C, with absorbance recorded at 0, 1, 2, 4, 6, 8, 10, 12, and 24 h to plot the curve of the absorbance versus time, from which the decomposition percentage of MnO_2_ NPs under different pH conditions were calculated.

To verify the ability of MnO_2_ to consume GSH, an equal volume of GSH aqueous solution (0.4 mg/mL) was added to an equal volume of MnO_2_@APS NPs with varying concentrations. The mixture was reacted at room temperature for 6 h, centrifuged at 12,000 rpm for 10 min, and 40 μL of the supernatant was collected to determine the remaining GSH using a GSH assay kit.

### 2.9 pH/GSH-dependent release of Mn^2+^ from MnO_2_@APS NPs

MnO_2_ nanoparticles catalyze the generation of Mn^2+^ in the tumor microenvironment in the presence of glutathione (GSH) and hydrogen ions (H^+^). The produced Mn^2+^ exhibits a T_1_ relaxation signal, with its concentration being directly proportional T_1_ transverse relaxation rate. To establish a calibration curve between the T_1_ relaxation rate and [Mn] concentration, MnCl_2_ solutions were used as standards. T_1_ relaxation times were measured using a VTMR20-010V-I (0.49 T) NMR analyzer. A series of MnCl_2_ working solutions were prepared at concentrations of 2, 1, 0.5, 0.25, 0.125, and 0.0625 mM, and their corresponding T_1_ relaxation times were recorded. The linear regression between [Mn] concentration and 1/T_1_ was fitted via Microsoft Excel 2016.

The pH 5.5 PBS containing 10 mM GSH and pH 7.4 PBS were employed to simulate the tumor microenvironment and normal tissue. MnO_2_@APS NPs nanoparticles were incubated in PBS at 37 °C under various conditions. Following centrifugation at 8000 rpm at 0, 1, 2, 4, 6, 8, 10, and 24 h, 1 mL of supernatant was collected and an equal volume of fresh release medium was supplemented. T_1_ relaxation time of collected samples were tested and [Mn] concentration in the samples was calculated to construct an *in vitro* Mn release profile.

### 2.10 Cellular uptake

4T1 cells were cultured to the logarithmic growth phase, then seeded in 24-well plates at a density of 1 × 10^5^ cells per well and cultured until reaching 75 % confluency. Then the cells were co-cultured with Squ/C6@APS NPs and Squ/C6@APS-IR820 NPs (C6 concentration of 0.5 μg/mL) for 1, 3, and 6 h, using the culture medium as a blank control. Then the medium was removed and cells were washed with 0.5 mL PBS, fixed with 4 % paraformaldehyde (PFA) for 20 min, rinsed twice with PBS. Cell nuclei were subsequently stained with 4,6-diamino-2-phenyl indole (DAPI) for 10 min, followed by three additional PBS washes. Finally, the cellular uptake of NPs was visualized using an inverted fluorescence microscope (TS2R Nikon, Japan).

For flow cytometry analysis, 4T1 cells were seeded in 6-well plates at a density of 5 × 10^5^ per well and cultured until reaching 75 % confluency. The cells were then co-cultured with Squ/C6@APS NPs and Squ/C6@APS-IR820 NPs (C6 concentration of 0.5 μg/mL) for 1 h, 3 h, 6 h respectively. Then, culture medium was discarded, the cells were washed three times with PBS, trypsinized, and collected for flow cytometry analysis on a flow cytometer (SH800S SONY, Japan).

To assess the potential involvement of OATPs in IR820 uptake, we pretreated 4T1 cells with 100 μM probenecid (an OATP 1/3 inhibitor [33]) or 50 μM doxorubicin (a general OATP inhibitor [34]) for 30 min prior to treatment with Squ/C6@APS-IR820 NPs or Squ/C6@APS NPs. Following 3 h of nanoparticles incubation, cells were washed three times with PBS and fixed with 4 % paraformaldehyde (PFA) for 20 min, followed by two additional PBS washes. Nuclei were then counterstained with DAPI (10 min) and washed three times with PBS. Cellular NPs uptake was visualized using a Nikon TS2R inverted fluorescence microscope (Japan).

### 2.11 Transwell invasion assays

The invasive capacity of 4T1 cells was assessed using matrigel-coated transwell chambers (6.5 mm diameter, 8 μm pore size; Corning). 2.5 × 10^4^ cells were seeded in the upper chambers, the complete medium was replaced with serum-free medium after cell attachment. After incubation for 12 h, saline (10 μL), Squ@APS-IR820 NPs (Squ, 0.075 μg/mL), MnO_2_@APS-IR820 NPs (MnO_2_, 3.75 μg/mL), and Squ@APS-IR820 NPs + MnO_2_@APS-IR820 NPs (Squ, 0.075 μg/mL, MnO_2_, 3.75 μg/mL) were added in the upper chambers. 12 h later, serum-free medium in the lower chambers were replaced with 600 μL of RPIM-1640 containing 10 % FBS. After incubation for 48 h, the invasive cells were fixed with 4 % paraformaldehyde and stained with 0.1 % crystal violet. The non-invasive cells remaining in the upper chamber were gently wiped away with a cotton-tipped applicator. Invasive cells in 3 random fields were calculated.

### 2.12 In vitro BMDC maturity assay

The femurs of C57BL/6 mice (6-week-old, female) was used to isolate bone marrow derived dendritic cells (BMDCs) using aforementioned methods [32] under sterile conditions. Briefly, both ends of the femur were cut to expose the bone marrow cavity. The bone marrow cells were then flushed out with ice-cold sterile PBS and filtered through a 70 μm cell strainer. Following red blood cell lysis, the cells were collected and cultured in 10 cm Petri dishes at a density of 1 × 10^7^ cells per dish in RPMI 1640 complete medium supplemented with GM-CSF (20 ng/mL) and IL-4 (10 ng/mL) for six days.

The obtained BMDCs were seeded in 6-well plates at a density of 1 × 10^6^ cells per well and cultured overnight. Subsequently, normal saline, PTX, Squ@APS-IR820 NPs, MnO_2_@APS-IR820 NPs, Squ@APS NPs + MnO_2_@APS NPs, and Squ@APS-IR820 NPs + MnO_2_@APS-IR820 NPs were incubated with BMDCs for 12 h at concentrations of 0.188 μg/mL Squ and 9.38 μg/mL MnO_2_. After incubation, BMDCs were gathered by centrifugation at 1500 rpm for 5 min, washed with pre-cooled PBS, resuspended in cell staining buffer for Trypan Blue staining and cell counting. CD16/32 antibody was used to block Fc receptors of cellular proteins for 10 min at room temperature to reduce nonspecific antibody binding. Cells were then sequentially stained with fluorescence-labeled antibodies (PerCP/Cy 5.5-*anti*-CD45, PE-anti-CD11c, FITC-anti-CD80, and APC-anti-CD86) in the dark at 4 °C for 30 min. Finally, 5 μL of DAPI (0.5 μg/mL) was added to each sample tube and incubated for 5 min to distinguish between live and dead cells. The percentage of DCs maturation was immediately detected on a flow cytometry (Beckman Cytoflex LX, USA).

### 2.13 Macrophages repolarization experiment

In order to comprehend the repolarization potential of TAMs following different treatments, bone marrow derived macrophages (BMDMs) were isolated. A method similar to BMDCs extraction was applied, except for the replacement of inducer (GM-CSF) with 50 ng/mL mouse M-CSF. The obtained BMDMs were cultured for 6 d, seeded in 6 well plates (cell density: 1 × 10^6^ cells per well), and stimulated with mouse IL-4 (20 ng/mL) for 12 h to differentiate these BMDMs into M2 macrophage. M1 macrophages were produced using stimulation with LPS (250 ng/mL) and stained with anti-CD86-PE antibody acting as single-stain sample. Then, M2 BMDMs were treated with PBS, PTX, Squ@APS-IR820, MnO_2_@APS-IR820, Squ@APS + MnO_2_@APS, Squ@APS-IR820 + MnO_2_@APS-IR820 respectively for 12 h. The cells were scraped and spined down to a pellet. The precipitated cells were resuspended in 200 μL cell staining buffer and stained by Zombie Violet (a viability dye), anti-CD45-APC (0.1 μg/10^6^ cells), anti-CD86-PE, and anti-F4/80-PerCP/Cy5.5. After being fixed by 4 % paraformaldehyde and permeabilized by permeabilization buffer, M2 macrophages were intracellularly stained with anti-CD206-FITC (0.1 μg/10^6^ cells), the macrophage phenotype was verified on a flow cytometry (Beckman Cytoflex LX, USA).

### 2.14 In vivo biodistribution in 4T1 tumor-bearing mice

A breast cancer mouse model was established by subcutaneously injecting 1.0 × 10^6^ 4T1 cells into the right axillary region of female Balb/c mice. Eight tumor-bearing mice with similar tumor volumes were randomly divided into two groups. The mice in each group were intravenously administrated with DiR-labeled Squ@APS (DiR:Squ:OA:APS = 0.2:0.02:1:8) or Squ@APS-IR820 NPs (Squ:OA:APS-IR820 = 0.02:1:8) respectively, at a dose of 15 μg/mL Squ in 0.2 mL. Fluorescently imaging was performed using an IVIS Imaging System (PerkinElmer) at 1, 2, 4, 8, 10, 24, 48 and 72 h post-injection (DiR-labeled Squ@APS NPs: λ_ex_ = 745 nm, λ_em_ = 800 nm; Squ@APS-IR820 NPs, λ_ex_ = 745 nm, λ_em_ = 820 nm).

One mouse from each group was euthanized at 8 h, while the remaining three mice per group were euthanized at 72 h. Tumors, hearts, livers, spleens, lungs, and kidneys were collected for *ex vivo* fluorescence imaging applying the IVIS Living Image system as described above. Semiquantitative fluorescence analysis was performed using Image analysis software to calculate the relative tumor targeting index (RTTI) according to Eq. (4):

(4) $$\text{RTTI}\%=F_{t}/F_{lv}\times 100\%$$

Where *F*_*t*_ and *F*_*lv*_ represents the average fluorescence intensity of tumor and liver.

### 2.15 In vivo anti-tumor therapy

The anti-tumor treatment was performed using a 4T1 tumor-bearing mouse model. In detail, female Balb/c mice (6–8 weeks old, weighing 22 ± 2 g) were housed for 5 d in a specific pathogen-free (SPF) animal facility at 25 °C with 70 ± 5 % relative humidity. A breast cancer mouse model was established by subcutaneously injecting 1.0 × 10^6^ 4T1 cells into the right axillary region of female Balb/c mice. When the tumor grows to approximately 150 mm^3^, the mice were randomly assigned to six groups (n = 6) and were intravenously administered the following treatments: normal saline, PTX injections (8 mg/kg), Squ@APS-IR820 NPs, MnO_2_@APS-IR820 NPs, Squ@APS NPs + MnO_2_@APS NPs, Squ@APS-IR820 NPs + MnO_2_@APS-IR820 NPs respectively (0.15 mg/kg for Squ, 7.5 mg/kg for MnO_2_) every two days (7 total doses). Tumor volume and body weight were monitored every 2 days. Tumor volume was calculated according to Eq. (5):

(5) $$V\left({mm}^{3}\right)=0.5\times xy^{2}\left({mm}^{3}\right)$$

where *x* is the longest diameter of the tumor, *y* is the shortest diameter of the tumor.

The day after the last dose, the mice were euthanized, and tissues (tumor, heart, liver, spleen, lung, and kidney) were dissected and weighed. Tumor inhibition rate (TIR) was calculated according to Eq. (6):

(6) $$TIR\left(\%\right)=\left(1-W_{e}/W_{c}\right)\times 100\%$$

Where W_e_ is the weight (volume) of tumor in experimental groups, W_c_ is the tumor weight (volume) in the negative control group treated with normal saline.

The liver, spleen indexes were determined by Eq. (7) and Eq. (8):

(7) $$Liver\;index=W_{LI}/W\times 100\%$$

(8) $$Spleen\;index=W_{sp}/W\times 100\%$$

Where W_*LI*_, W*sp* is the weight of liver and spleen, *W* is the body weight.

On the day following the final dose, mice were anesthetized and blood was collected from the retro-orbital sinus. Following 1-h coagulation at 4 °C, blood samples were centrifuged at 3500 rpm for 10 min to obtain serum. Liver function was assessed by measuring serum alanine aminotransferase (ALT) and aspartate aminotransferase (AST) levels, while renal function was evaluated through urea and creatinine (CRE) quantification using an automated biochemical analyzer (AU480, Beckman Coulter, Japan).

Histological analysis was performed by staining tumor tissues with hematoxylin and eosin (H&E). TUNEL assay was used to evaluate tumor cell apoptosis.

### 2.16 Anti-tumor immune responses of the MnO_2_ NPs based chemoimmunotherapy

Three tumor-bearing mice were randomly selected from Squ@APS-IR820, MnO_2_@APS-IR820, and Squ@APS-IR820+MnO_2_@APS-IR820 groups after the last dose. The tumor, spleen samples from each group were dissected and stored in cold RPMI 1640 medium. Tumor samples were digested with collagenase Ⅳ, hyaluronidase, and DNAse Ⅰ for 1 h at 37 °C. Single-cell suspensions of tomors were prepared through Percoll density gradient centrifugation. Splenic single-cell suspensions were obtained using grinding and filtering according to routine protocols (Bio-protocol, USA). After blocking with anti-CD16/32 antibody for 15 min at room temperature, single-cell suspensions were stained with fluorescently labeled antibodies according to the manufacturer's instructions. Flow cytometry ((Cytoflex LX Beckman, USA)) was used to analyze cellular populations. Specifically, tumor single-cell suspensions were used to analyze the percentage of CD45^+^CD3^+^CD4^+^ T cells, CD45^+^CD3^+^CD8^+^ T cells, and CD45^+^CD11b^+^Ly6C^+^ MDSCs. Splenic single-cell suspensions were used to calculate the percentage of CD45^+^CD3^+^CD4^+^ T cells, CD45^+^CD3^+^CD8^+^ T cells, CD45^+^ CD11c^+^Gr1(Ly6G + Ly6C)^−^CD80^+^CD86^+^ DCs, and CD45^+^CD3^-^B220^+^ B cells.

### 2.17 Statistical analysis

All of data was analyzed with Microsoft Excel 16 and Graphpad Prism 10.1.2. One-way ANOVA test was used to evaluate difference among multiple groups, and Welch's *t*-test was conducted to test for differences between two groups. P value of less than 0.05 was considered statistically significant.

## 3 Results and discussion

### 3.1 Preparation and characterization of MnO_2_ NPs

MnO_2_ NPs could be synthetized through mild reaction of KMnO_4_ with reducing reagents [35] such as 2-(Nmorpholino) ethanesulfonic acid (MES) [36] and cationic polyelectrolyte poly(allylamine hydrochloride) (PAH) [37] et al. In this study, we developed a novel MnO_2_ NPs preparation method, which is environmental-friendly, with low cost comparing to the reported strategies. In brief, KMnO_4_ aqueous solution were mixed with anhydrous ethanol and stirred at room temperature overnight. The concentration of KMnO_4_ solution, the volumetric ratio of KMnO_4_ aqueous solution/ethanol, the stirring speed, and the reaction temperature were adjusted to achieve optimal particle size and polydispersity index (PDI) of the resultant MnO_2_ nanoparticles (Table S1 and Fig. S2). When a 3 mg/mL KMnO_4_ aqueous solution was mixed with three volumes of ethanol and stirred at 400 rpm at room temperature overnight, the resulting MnO_2_ nanoparticles exhibited minimal particle size and polydispersity index (PDI) values, achieving a mean yield of 82.2 %. Thus, all MnO_2_ nanoparticles used in the subsequent experiments were prepared under these conditions. The resultant MnO_2_ NPs displayed an average particle size of 143.2 ± 0.058 nm, a PDI of 0.086 ± 0.005 and a surface Zeta potential of −36.1 ± 1.24 mV.

**Fig. 1:**
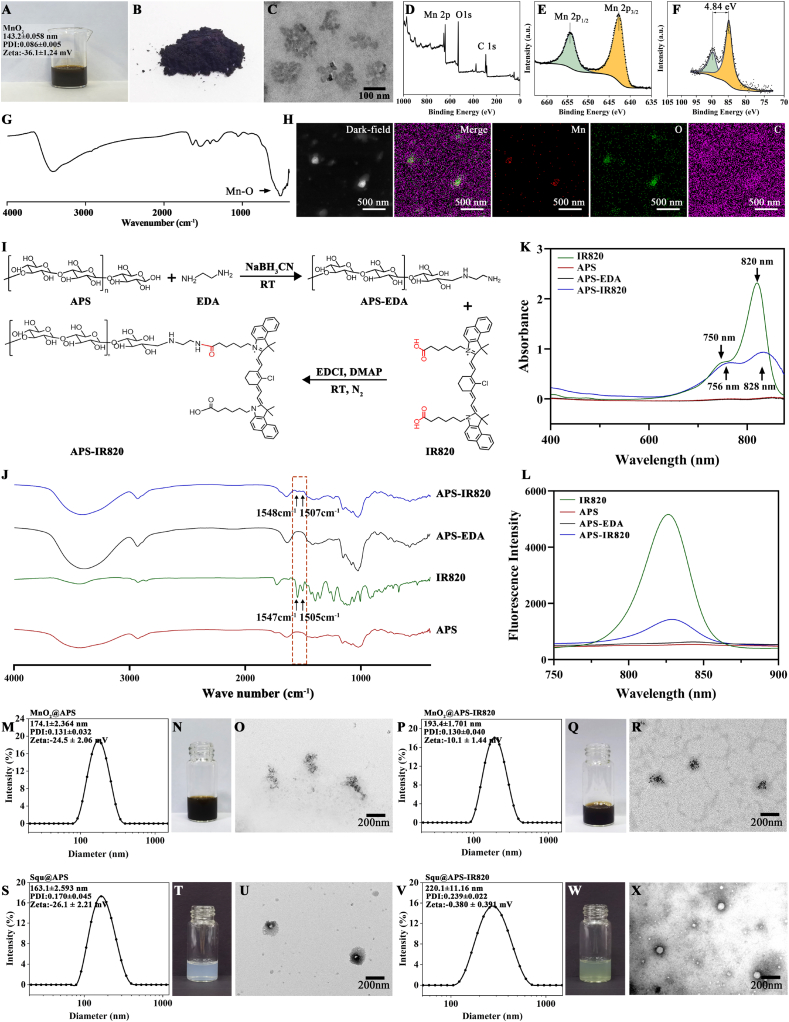
Synthesis and characterization of materials and nanoparticles. (A) Photograph of MnO_2_ NPs dispersed in DI water. (B) Lyophilized powder of MnO_2_ NPs. (C) TEM of MnO_2_ NPs. (D) XPS spectra of MnO_2_ NPs. (E) High resolution XPS spectra of Mn 2p. (F) High resolution XPS spectra of Mn 3s. (G) FT-IR spectra of MnO_2_ NPs. (H) Dark-field STEM and corresponding elemental mapping of MnO_2_@APS-IR820 NPs. (I) Scheme for synthesis of APS-EDA and APS-IR820. (J) FT-IR spectra of APS, IR820, APS-EDA, APS-IR820. (K) Absorption spectra of IR820 in methanol, APS, APS-EDA, APS-IR820 in DI water. (L) Fluorescence spectrum of APS-IR820 after 710 nm excitation in mixture of DMSO and water (3:7 v/v) solution. (M) Particle size distribution curves, appearance (N) and TEM images (O) of MnO_2_@APS NPs. (P) Particle size distribution curves, appearance (Q) and TEM images (R) of MnO_2_@APS-IR820 NPs. (S) Particle size distribution curves, appearance (T) and TEM images (U) of Squ@APS NPs. (V) Particle size distribution curves, appearance (W) and TEM images (X) of Squ@APS-IR820 NPs.

As seen in Fig. 1A and B, the aqueous solution of MnO_2_ NPs was brown, and the dark brown powder was obtained after lyophilization for subsequent studies. Transmission electron microscopy (TEM) demonstrated a spherical morphology, with individual particles approximately 20 nm in size, forming clusters around 150 nm (Fig. 1C). The chemical composition of MnO_2_ NPs was confirmed by X-ray photoelectron spectroscopy (XPS) to contain both O and Mn elements (as shown in Fig. 1D). The Mn 2p spectrum (Fig. 1E) revealed spin-orbit binding energy levels of 642.4 eV (Mn 2p^3/2^) and 654.0 eV (Mn 2p^1/2^), indicating Mn^4+^ oxidation state in the resultant nanoparticles. The Mn oxidation states were further characterized by analyzing the Mn 3s peak (Fig. 1F), which exhibited two multiplet-split components resulting from the coupling between non-ionized 3s electrons and 3d valence-band electrons. The oxidation state can be determined by measuring the energy separation of these multiplet peaks (Cited from XPS knowledge view of Thermo Avantage). The experimentally determined ΔE value of the synthesized MnO_2_ NPs was 4.84 eV, consistent with the reference ΔE value for Mn^4+^ in MnO_2_ (4.7 eV) based on XPS analysis using Thermo Avantage software. The FT-IR spectrum showed a characteristic absorption peak at ∼520 cm^−1^, corresponding to the Mn–O bond Fig. 1G. These results confirm the successful synthesis of MnO_2_ nanoparticles.

### 3.2 Synthesis and characterization of APS-IR820

IR820, a heptamethine cyanine dye, shares similar structure and function with Cy7 dyes, which is a near-infrared fluorescent molecule [25]. The meso-chlorine (Meso-Cl) group of IR820 can be displaced by free thiol in albumin at ^34^Cys site in blood, the resulting albumin adducts accumulate in tumors via the EPR effect. Furthermore, Organic Anion Transporting Polypeptide (OATPs) contribute to the uptake of tumor seeking dyes by cancer cells [27]. Therefore, IR820 is considered as a tumor-seeking dye. IR820 has strong tumor targeting and long-term tumor retention properties, according to our previous study (Fig. S6). IR820 dihexanoic acid was used in this research, abbreviated as IR820 in this article, the chemical structure of which was shown in Fig. 1I.

IR820 was conjugated with APS to create a multifunctional pharmaceutical excipient for *in vitro* and *in vivo* imaging and tumor targeting, while also stabilizing MnO_2_ NPs and encapsulating Squ. The semi-acetal structure in the terminal monosaccharide residues of APS exists in an equilibrium between ring-opening and ring-closing forms in solution [38]. This tautomerism makes it possible for existence of aldehyde groups in the solution, which is prone to form Schiff base with the primary amino group of ethylenediamine (EDA). Then, Schiff base was reduced to amine by NaBH_3_CN. Then the amino group of the resultant APS-EDA (ammoniated polysaccharide) reacted with the carboxyl group of IR820 to yield APS-IR820 (Fig. 1I). Unreacted IR820 was efficiently removed through dialysis and ethanol precipitation. After lyophilization, APS-IR820 was obtained as a dark green, water-soluble powder with a total yield of 77.3 %.

Characterization confirmed the absence of free IR820, and the conjugate exhibited complete solubility in deionized water, forming a stable dark green solution.The structure of APS-IR820 were confirmed using Fourier transform infrared spectroscopy, UV–VIS spectroscopy, and fluorescence spectroscopy. As seen in Fig. 1J, IR820 showed a characteristic absorption band at 1547 and 1505 cm^−1^ corresponding to benzene ring skeleton vibration in its FT-IR spectrum, which were absent in the FT-IR spectra of APS and APS-EDA, but present at 1548 and 1507 cm^−1^ in the FT-IR spectrum of APS-IR820, indicating the successful introduction of IR820 into APS macromolecule.

VIS spectra of APS, IR820, APS-EDA, and APS-IR820 was shown in Fig. 1K. APS and APS-EDA have no characteristic absorption in the range of 400–900 nm, while APS-IR820 showed a characteristic peaks at round 756 nm and 828 nm in DI water, corresponding to those of IR820, this also evidenced the successful labelling of APS with IR820.

The fluorescence emission spectrum of APS, IR820, APS-EDA, and APS-IR820 after 710 nm excitation was shown in Fig. 1L. The maximum emission wavelength of APS-IR820 was at around 833 nm, which is close to the maximum emission wavelength of IR820 itself, while APS and APS-EDA produced no fluorescence emission spectra.

Therefore, it can be confirmed that APS-IR820 has been successfully synthesized.

A standard curve for the concentration-fluorescence intensity of IR820 in DMSO-DI (3:7 v/v) solution was established using fluorescence spectrophotometry (λ_ex_ = 710 nm, λ_em_ = 833 nm), with the standard curve equation being Y = 460.9X + 591.7 (R^2^ = 0.9993, linear range 1 μg/mL-10 μg/mL). The average fluorescence intensity of APS-IR820 DMSO-DI aqueous solution at 1 mg/ml was measured three times and the IR820 content in APS-IR820 was calculated to be 3.83 mg/g according to the above standard curve equation.

### 3.3 Preparation and characterization of nanoparticles

#### 3.3.1 Preparation and characterization of MnO_2_@APS and MnO_2_@APS-IR820 NPs

Bare MnO_2_ NPs exhibited poor stability in physiological media, tending to aggregate and precipitate in PBS or normal saline, while also demonstrating significant particle size enlargement in 5 % glucose solution (Fig. S2). Hydrophilic polymers were usually applied to improve the stability of MnO_2_ NPs, such as poly(acrylic acid) (PAA) [22], PEG [23], polylysine (PLL) [9] et al. In this study, APS was modified at the surface of MnO_2_ NPs to increase their physical stability, due to the good biocompatibility, safety, and weak anti-tumor activity of APS. The resultant MnO_2_@APS NPs were dark brown colloidal system with an average particle size of 174.1 ± 2.364 nm, a PDI of 0.131 ± 0.032, a zeta potential of −24.5 ± 2.06 mV, and irregular clusters morphology observed by TEM (Fig. 1M–O). MnO_2_@APS-IR820 NPs were also dark brown with a slightly larger particle size of 193.4 ± 1.701 nm, a PDI of 0.130 ± 0.040 (Fig. 1P–R), and a zeta potential of −10.1 ± 1.44 mV. The composition of MnO_2_@APS-IR820 NPs was further confirmed by the STEM-based elemental mapping (Fig. 1H).

#### 3.3.2 Preparation and characterization of Squ@OA-APS and Squ@OA-APS-IR820 NPs

Squ is a highly lipophilic long-chain molecule with poor water solubility (∼7.5 μg/mL), which limits its *in vivo* delivery. To address this challenge, Squ was formulated into nanoparticles, and in this study its anti-tumor efficacy both as a monotherapy and in combination with MnO_2_@APS NPs or MnO_2_@APS-IR820 NPs was evaluated.

**Fig. 2:**
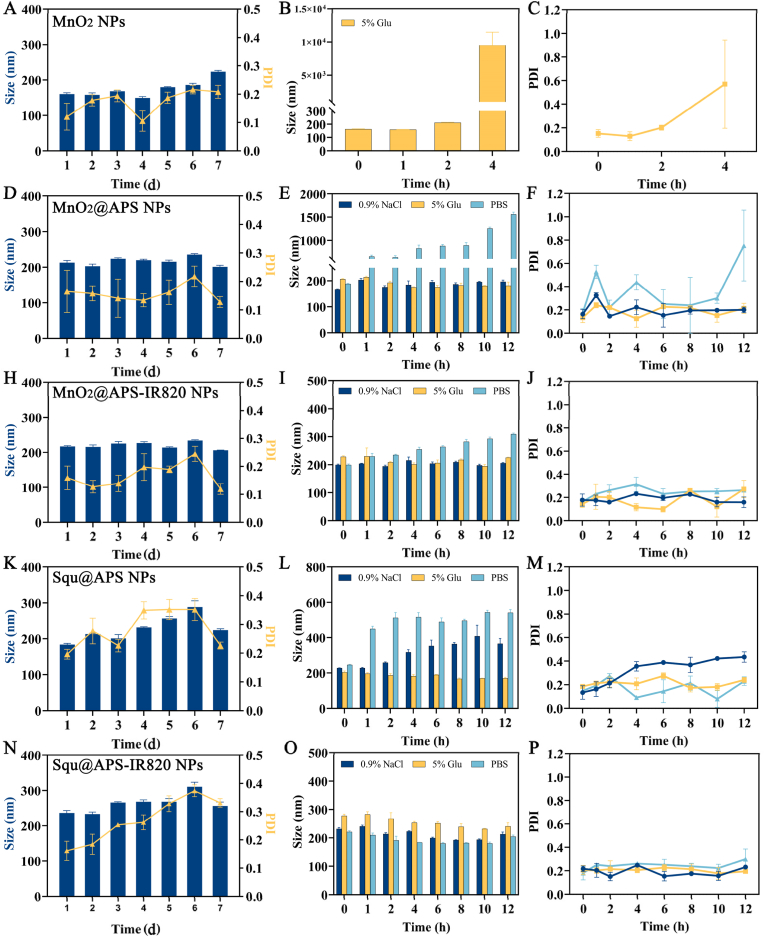
Stability of various nanoparticles. The mean particle size and PDI of MnO_2_ NPs during 7 d storage on shelf (A), the mean particle size change (B) and PDI change (C) of MnO_2_ NPs when incubated in 5 % glucose at 37 °C; The mean particle size and PDI of MnO_2_ @APS NPs during 7 d storage on shelf (D), the mean particle size change (E) and PDI change (F) of MnO_2_@APS NPs when incubated in 0.9 % NaCl, PBS and 5 % glucose at 37 °C; The mean particle size and PDI of MnO_2_ @APS-IR820 NPs during 7 d storage on shelf (H), the mean particle size change (I) and PDI change (J) of MnO_2_@APS-ID820 NPs when incubated in 0.9 % NaCl, PBS and 5 % glucose at 37 °C; The mean particle size and PDI of Squ@APS NPs during 7 d storage on shelf (K), the mean particle size change (L) and PDI change (M) of Squ@APS NPs when incubated in 0.9 % NaCl, PBS and 5 % glucose at 37 °C; The mean particle size and PDI of Squ@APS-IR820 NPs during 7 d storage on shelf (N), the mean particle size change (O) and PDI change (P) of Squ@APS-IR820 NPs when incubated in 0.9 % NaCl, PBS and 5 % glucose at 37 °C.

When APS alone was used to prepare Squ nanoparticles by dropwise addition of a Squ/methanol solution into APS solution under stirring (followed by methanol removal), the resulting Squ@APS nanoparticles exhibited small and uniform particle sizes but showed significant precipitation after 5 h. However, when a small amount of oleic acid (OA) was added into the Squ/methanol solution, the resultant Squ@APS NPs were much more stable. The mass ratio of Squ@APS-IR820 was determined at 0.2:1:8 (Squ: OA: APS-IR820) by formulation optimization. Squ@APS and Squ@APS-IR820 has good storage stability with the mean particle size and PDI slightly increased within 7 days’ storage on shelf (Fig. 2K, N). The presence of OA may increase the elasticity of nanoparticles and help them enter tumors. Oleic acid (OA) is a long-chain unsaturated fatty acid and is commonly used as surfactant. The double bond in OA molecules can cause spatial structure bending, create spatial barriers, and hinder entanglement of adjacent chains, thus can be applied to prevent aggregation of nanoparticles [39]. Squ@APS-IR820 NPs were supposed to have tumor-targeting potential due to encapsulation of APS-IR820.

Squ@APS NPs and Squ@APS-IR820 NPs showed an average particle size of 163.1 ± 2.593 nm (PDI = 0.170 ± 0.045) (Figs. 1S) and 220.1 ± 11.16 nm (PDI = 0.239 ± 0.022) (Fig. 1V) respectively. The zeta potentials were −26.1 ± 2.21 mV and −0.380 ± 0.391 mV respectively. Squ@APS NPs show light blue opalescence (Fig. 1T) and exhibits a spheric morphology in TEM image (Fig. 1U). Meanwhile, Squ@APS-IR820 NPs presented green opalescence (Fig. 1W), and displayed spherical morphology in TEM image (Fig. 1X). DLC and EE of Squ @ APS NPs is 2.59 % and 90.04 % %, and DLC and EE of Squ @ APS-IR820 is 2.91 % and 92.0 %. The nanoparticles can fully reach the administered dose due to the dosage of Squ being as low as 0.15 mg/kg.

### 3.4 Stability

MnO_2_ NPs, MnO_2_@APS NPs, MnO_2_@APS-IR820 NPs all showed presentable stability on shelf at room temperature (Fig. 2A, D and H), but their stability in physiological media were quite different. MnO_2_ NPs displayed poor stability in both PBS and 0.9 % NaCl, exhibiting immediate aggregation and precipitation (Fig. S2). In 5 % glucose solution, MnO_2_ NPs maintained relatively stable particle sizes for the first 4 h, followed by significant size increase thereafter (Fig. 2B and C). Overall, the unmodified MnO_2_ NPs exhibited very limited stability under physiological conditions.

MnO_2_@APS NPs were very stable in saline and 5 % glucose with small mean particle size and a PDI of about 0.2 during 12 h' incubation at 37 °C, while their mean particle size continuously increased from 200 nm to about 1500 nm in PBS (Fig. 2E and F). It seemed that MnO_2_@APS NPs could be dispersed in saline or 5 % glucose solution for intravenous injection. As for MnO_2_@APS-IR820 NPs, probably due to that the π-π stacking between the unsaturated conjugate system of IR820 molecules strengthened the interior structure of MnO_2_@APS-IR820 NPs, making MnO_2_@APS-IR820 NPs more stable with limited mean particle size changes and limited PDI changes during the 12 h’ incubation in 5 % glucose solution, normal saline and PBS (Fig. 2I and J).

The same phenomenon was observed in Squ@ APS NPs and Squ@APS-IR820 NPs too. Both nanoparticles demonstrated good stability on shelf within 7 days and good stability in 5 % glucose solution, but Squ@APS NPs displayed significant particle size and PDI increase in normal saline and PBS (Fig. 2L and M) while Squ@APS-IR820 NPs were quite stable with slight changes in particle size and PDI (Fig. 2O and P).

### 3.5 The degradation and Mn^2+^ release of MnO_2_@APS NPs in response to pH/GSH

**Fig. 3:**
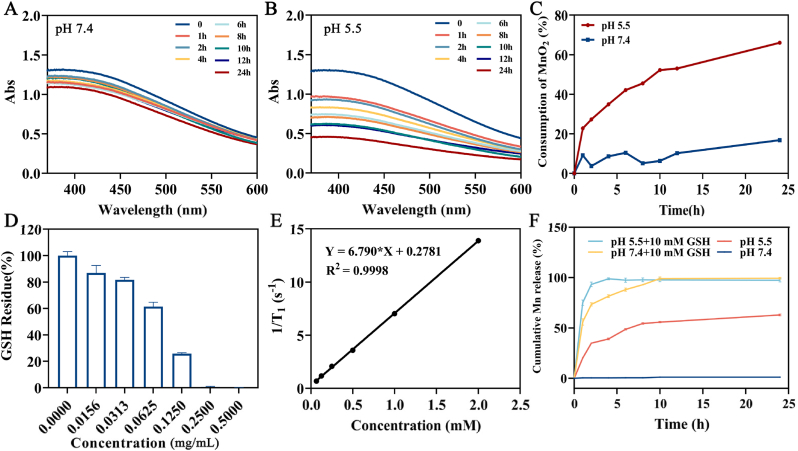
The degradation and Mn^2+^ release of MnO_2_@APS NPs in response to pH/GSH. The absorbance of MnO_2_@APS NPs in pH 7.4 (A) and pH 5.5 (B) at different time point. The consumption of MnO_2_ of MnO_2_@APS NPs at different time point (C). GSH residue rate after mixed with different concentration of MnO_2_@APS NPs for 6 h (D). The linear relationship between T_1_ transverse relaxation rate and [Mn] concentration (E). The cumulative release of Mn^2+^ from MnO_2_@APS NPs at pH 5.5, pH 7.4, pH 5.5 + 10 mM GSH, pH 7.4 + 10 mM GSH (F).

The responsive degradation of MnO_2_@APS NPs could be assessed by the decrease of MnO_2_-characteristic absorbance [23] as MnO_2_ reacts with H^+^ in the weakly acidic tumor environment and produces Mn^2+^. As seen in Fig. 3A, the UV absorption of MnO_2_@APS NPs reduced by 16.8 % at pH 7.4 within 24 h. In comparison, at pH 5.5, it reduced by 22.8 % in 1 h and 66.0 % in 24 h (Fig. 3B and C), indicating that MnO_2_ based NPs degraded in an acid responsive and time-dependent manner.

The degradation and transformation of MnO_2_@APS NPs into Mn^2+^ was also a GSH-consuming process. process. For this study, a GSH standard curve was established to quantify GSH concentration in the solution with the linear equation given as Y = 0.002167X - 0.006727 (R^2^ = 0.9975, in a linear range of 12.5 μg/mL-300 μg/mL). When 400 μg/mL of GSH was mixed with the same volume of different concentration of MnO_2_@APS NPs for 6 h’ reaction, it could be seen that, the higher concentration of MnO_2_, the less GSH left, and 0.25 mg of MnO_2_@APS (calculated by MnO_2_) NPs completely consumed 400 μg of GSH (Fig. 3D).

As Mn^2+^ generated from MnO_2_ could act as STING activator, Mn release kinetics under TME-mimicking conditions was evaluated. The Mn^2+^ concentration shows a linear correlation with 1/T_1_ (shown in Fig. 3E), following equation Y = 6.790X + 0.2781 (R^2^ = 0.9998, in a linear range from 0.0625 mM to 2.00 mM). As shown in Fig. 3F, less than 5 % Mn release was observed at pH 7.4 in the absence of GSH, while the cumulative Mn^2+^ release from MnO_2_@APS NPs reached approximately 62.9 % within 24 h at pH 5.5 without GSH. The addition of GSH significantly accelerated Mn release. In the presence of 10 mM GSH, the complete Mn release from MnO_2_@APS NPs was achieved at the 4th hour hours at pH 5.5 and at the 10th hour at pH 7.4. These results demonstrate that MnO_2_@APS NPs exhibit both GSH- and pH-responsive release pattern, with GSH playing a crucial role in accelerating Mn release.

The tumor microenvironment is characterized as weak acidity and high GSH level. It has reported that in the acidic region of TME, gene expression programs related to invasion and metastasis were extensively activated. Meanwhile, acidic TME increases the instability and heterogeneity of tumor cell genomes, leading to the formation of more resistant tumor variants [40]. GSH is assigned to maintain cellular redox homeostasis and associated with tumor occurrence, metastasis, and drug-resistance [41]. Meanwhile, GSH can neutralize ROS and reduce the therapeutic effects of phototherapy, radiotherapy, etc., making GSH targeting a new therapeutic target. MnO_2_-based nanoparticles can generate Mn^2+^ in response to the weak acidity and high GSH levels of the tumor microenvironment (TME). By consuming excess H^+^ and GSH, along with activating STING and exerting the immunoregulatory effects of Mn^2+^, these nanoparticles show promise in remodeling the immunosuppressive TME and enhancing the anti-tumor efficacy of chemotherapies.

### 3.6 Cellular uptake of Squ@APS NPs and Squ@APS-IR820 NPs

**Fig. 4:**
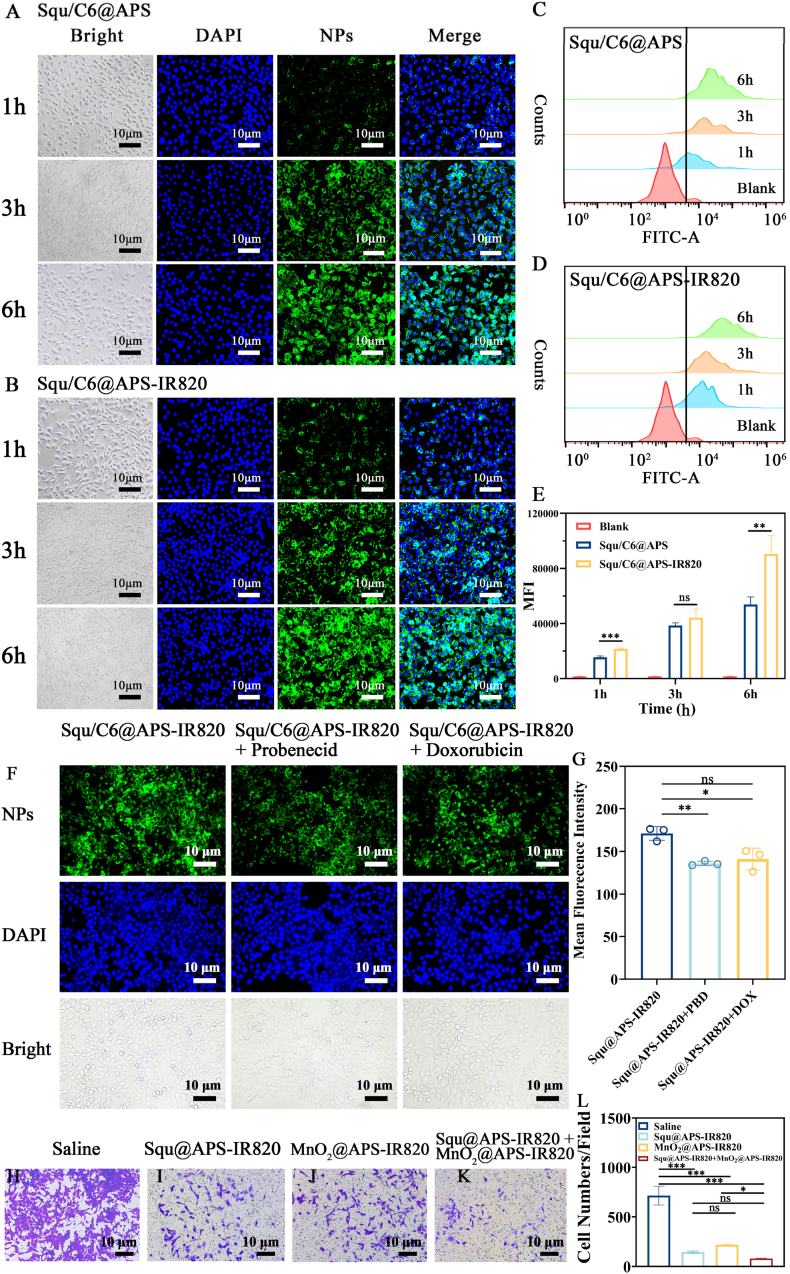
Cellular uptake of Squ@APS NPs and Squ@APS-IR820 NPs and transwell invasion assays. Fluorescence image of 4T1 cells co-cultured with Squ/C6@APS (A) and Squ/C6@APS-IR820 (B) at 1 h, 3 h, and 6 h, respectively. Blue and green represent DAPI and C6 fluorescence, which stand for the position of cell nucleus and NPs. The endocytosis of Squ/C6@APS (C) and Squ/C6@APS-IR820 (D) by 4T1 cells was visualized by flow cytometry. Blank represents cells treated with the same volume of medium, which was used as a control (Red). The mean fluorescence intensity (MFI) value (E) of 4T1 cells co-cultured with Squ/C6@APS and Squ/C6@APS-IR820NPs for 1 h, 3 h, and 6 h. Fluorescence image (F) and the mean fluorescence intensity (MFI) value (G) of 4T1 cells co-cultured with Squ/C6@APS-IR820 NPs for 3 h pretreated with 100 μM of probenecid or 50 μM of doxorubicin for 30 min. Invasion of 4T1 cells treated with saline (H), Squ@APS-IR820 NPs (I), MnO_2_@APS-IR820 (J), and Squ@APS-IR820 NPs + MnO_2_@APS-IR820 (K). Invasive cells in every field (L). Data are presented as means ± standard deviation (SD). Differences among groups were evaluated by one-way ANOVA analysis. ∗∗∗*p* < 0.001, ∗∗*p* < 0.01, and ∗*p* < 0.05. ns, no significant difference. (For interpretation of the references to colour in this figure legend, the reader is referred to the Web version of this article.)

To verify that APS-IR820-encapsulated nanoparticles perform a higher tumor cell uptake, Squ@APS NPs and Squ@APS-IR820 NPs were fluorescently labeled with coumarin 6 (C6) for cellular uptake analysis in 4T1 cells. The concentration of Squ used was kept well below its IC50 (Fig. S3 A, B) to ensure 4T1 cell viability. Both nanoparticles were incubated with 4T1 cells for 1 h, 3 h, and 6 h, followed by observation and analysis using an inverted fluorescence microscope. The green fluorescence intensity (corresponding to the NPs) increased significantly with extended incubation time, indicating that the time-dependent uptake of both nanoparticles by 4T1 cells (Fig. 4A and B). Notably, 4T1 cells incubated with Squ/C6@APS-IR820 NPs displayed stronger fluorescence intensity than those incubated with Squ/C6@APS NPs at each time point (Fig. 4A and B), which was more clearly evidenced by the flow cytometry (Fig. 4C and D) and their semi-quantitative analysis (Fig. 4E). These results indicate that APS-IR820 modification dramatically enhanced internalization of Squ nanoparticles by 4T1 cells in comparison with APS modification.

This observation aligns with previously reported findings [25] that tumor-seeking dyes are preferentially taken up by cancer cells through overexpressed organic anion-transporting polypeptides (OATPs) on their membranes. To investigate OATP's role in IR820 uptake by 4T1 cells, we pretreated the cells with two established OATP inhibitors: probenecid and doxorubicin. As shown in Fig. 4F, G, 20.52 % (*p* < 0.01) and 17.54 % (*p* < 0.05) accumulation of Squ/C6@APS-IR820 in 4T1 cells was inhibited after being incubated with probenecid and doxorubicin. While this suppressive trend was not significant in the uptake of Squ/C6@APS NPs under the same treatment (Fig. S4). The outcome demonstrated that OATP are partially involved in Squ/C6@APS-IR820 cellular uptake.

### 3.7 Transwell invasion assays

The Transwell invasion assays demonstrated potent inhibition of 4T1 cell invasion by both Squ@APS-IR820 (143 ± 10 cells/field, *p <* 0.0001) and MnO_2_@APS-IR820 (214 ± 6 cells/field, *p <* 0.0001) compared to the saline control (714 ± 96 cells/field). Notably, combination therapy with Squ@APS-IR820 + MnO_2_@APS-IR820 synergistically enhanced this anti-invasive effect, reducing penetrated cells to 77 ± 5 cells/field (*p <* 0.0001 vs saline), shown in Fig. 4H–L. Results demonstrate the anti-invasive potential of Squ- and MnO_2_-based delivery strategies against 4T1 cells.

### 3.8 In vitro BMDC maturity assay

Dendritic cells (DCs), as the most powerful APCs, bridge between innate and adaptive immunity. Untreated dendritic cells are in an immature state, expressing low levels of co-stimulatory factors and adhesion factors. However, immature DCs have strong antigen phagocytic ability and can be stimulated to differentiate into mature DCs. Mature DCs express at high levels of co-stimulatory factors and adhesion factors, which further present the contacted antigens to T lymphocytes to stimulate adaptive immunity. Therefore, DC maturation is an important indicator for immune activation.

**Fig. 5:**
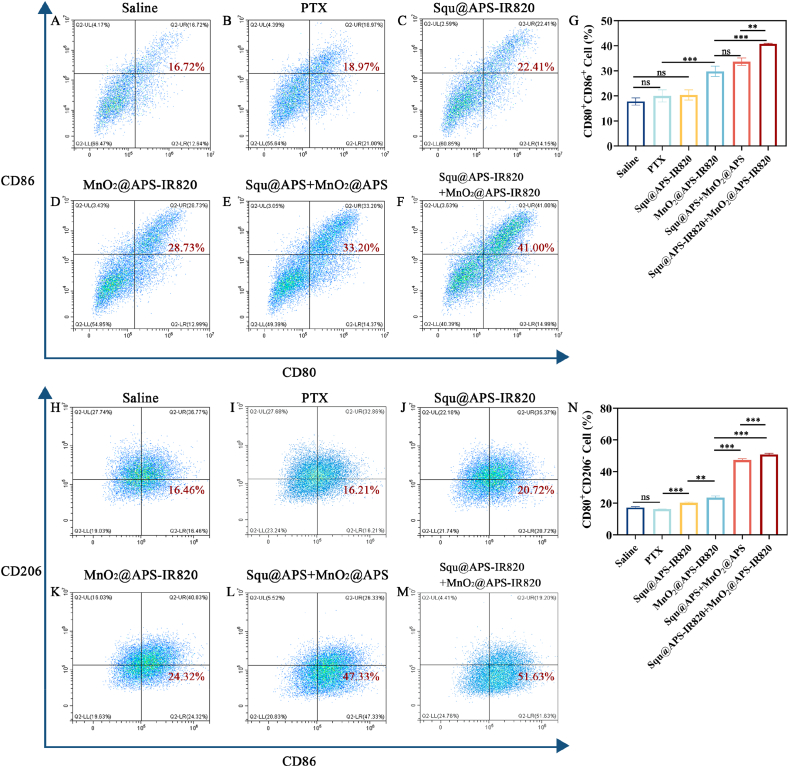
*In vitro* BMDC Maturity and BMDM Repolarization Assay. Representative flow cytometry graph of CD80^+^ CD86^+^ cells in BMDCs after treated with normal saline (A), PTX injections (B), Squ@APS-IR820 NPs (C), MnO_2_@APS-IR820 NPs (D), Squ@APS NPs + MnO_2_@APS NPs (E), and Squ@APS-IR820 NPs + MnO_2_@APS-IR820 NPs (F). Percentages of CD80^+^CD86^+^ cells (G). Flow cytometry chart of CD86^+^CD206^-^ cells (H–M) and quantification of the proportion (N) of CD86^+^CD206^-^ BMDMs after being incubated with different treatments (n = 3). Differences among groups were calculated by one-way ANOVA analysis. ∗*p* < 0.05, ∗∗*p* < 0.01, and ∗∗∗*p* < 0.001. ns, no significance.

In order to evaluate the immune activation effects of different formulations, bone marrow-derived dendritic cells were extraced [32] and incubated with normal saline, PTX injections, Squ@APS-IR820 NPs, MnO_2_@APS-IR820 NPs, Squ@APS NPs + MnO_2_@APS Nps, Squ@APS-IR820 NPs + MnO_2_@APS-IR820 NPs respectively to assess their DCs maturation ability by detecting the percentage of mDCs expressing both CD80 and CD86 on the surface. The gating strategy for flow cytometry analysis of BMDC was shown in Fig. S5. The PTX (Fig. 5B) and Squ@APS-IR820 (Fig. 5C) could induced an average DCs maturation percentage of 19.96 % and 20.37 % respectively, which were comparable to normal saline group (Fig. 5A)(17.78 %). However, after the treatment of MnO_2_@APS-IR820 NPs (Fig. 5D), Squ@APS NPs + MnO_2_@APS NPs (Fig. 5E), and Squ@APS-IR820 NPs + MnO_2_@APS-IR820 NPs (Fig. 5F), the proportion of CD80^+^CD86^+^mature BMDCs reached 29.78 %, 33.65 %, and 40.72 % (Fig. 5G) respectively.

This outcome indicated that MnO_2_ based nanoparticles play a major role in promoting BMDCs maturation, and the addition of Squ in the prescription has certain synergistic effects in this regard. Squ@APS-IR820 NPs + MnO_2_@APS-IR820 NPs treatment induced stronger BMDCs activation than Squ@APS NPs + MnO_2_@APS NPs treatment (*p* < 0.01), this demonstrated that IR820 modification also contributed to DC maturation by enhancing tumor targeting.

### 3.9 Macrophages repolarization experiment

Macrophages can typically be classified into two subgroups: M1-type (classically activated macrophages) and M2-type (alternatively activated macrophages). Tumor-associated macrophages (TAMs) predominantly exhibit an M2-like phenotype and secrete immunosuppressive factors, serving as key regulators of the immunosuppressive tumor microenvironment (TME) that promote immune escape and tumor progression. M1 type TAMs can produce some pro-inflammatory factors, such as IL-12 and tumor necrosis factor-α (TNF-α), which can kill tumor cells and activate other immune cells, such as NK cells and T cells [42]. The phenotype of TAMs can be reshaped, therefore, repolarizing TAMs from M2 to M1 is considered a promising therapeutic strategy for tumor treatment [43]. To evaluate the macrophage-repolarizing capability of each treatment regimen, we analyzed bone marrow-derived macrophage (BMDM) subtypes using flow cytometry. CD206 is selected as a marker for M2 TAMs (F4/80^+^CD206^+^), while CD86 (F4/80^+^CD86^+^) serves as a marker for M1 TAMs.

M0 BMDMs isolated from mouse bone marrow were induced to M2 type and treated with normal saline, PTX injections, Squ@APS-IR820 NPs, MnO_2_@APS-IR820 NPs, Squ@APS NPs + MnO_2_@APS NPs, Squ@APS-IR820 NPs + MnO_2_@APS-IR820 NPs respectively and their surface biomarker was testified by flow cytometry. The gating strategy for flow cytometry analysis of BMDM was shown in Fig. S5. All of the above nanoparticles can induce transformation of M2 BMDMs to M1 BMDMs to varying degrees (Fig. 5N) with the exception of normal saline (Fig. 5H) and PTX injections (Fig. 5I). Squ@APS NPs + MnO_2_@APS NPs (Fig. 5L), Squ@APS-IR820 NPs + MnO_2_@APS-IR820 NPs (Fig. 5M) exhibit stronger repolarization effects than Squ@APS-IR820 NPs (Fig. 5J) and MnO_2_@APS-IR820 NPs (Fig. 5K), indicating the combination strategy was more effective than the single treatment.

### 3.10 In vivo biodistribution in 4T1 tumor-bearing mice

Cellular uptake assay demonstrated that 4T1 cells uptook significantly more Squ@APS-IR820 NPs than Squ@APS NPs, primarily due to the tumor-seeking effects of IR820 molecule. The tumor-targeting capability of IR820 was validated through biodistribution studies. As shown in Fig. S6A, fluorescence was observed in tumors as early as 1 h post intravenous administration, peaking at 48 h. At 72 h, the average fluorescence intensity in *ex vivo* tumors was approximately 3-fold higher than in liver tissue (Fig. S6B and C), demonstrating both tumor-targeting specificity and prolonged retention of IR820. This targeting effect could be effectively transferred to the APS through chemically conjugating APS and IR820, endowing APS-IR820-based nanoformulations with dual advantages: tumor-specific accumulation and retention. Subsequent *in vivo* biodistribution studies were conducted to verify the tumor-targeting performance of APS-IR820 encapsulating nanoparticle systems.

**Fig. 6:**
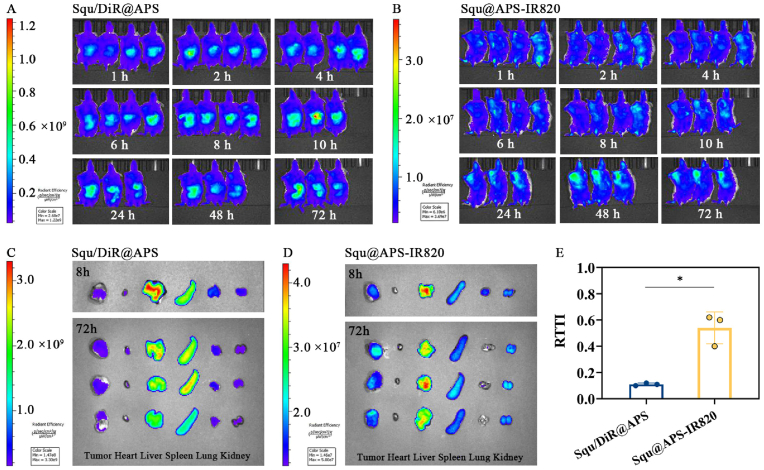
Biodistribution of Squ/DiR@APS NPs and Squ@APS-IR820 NPs in 4T1 tumor-bearing mice. Fluorescence images of dynamic bio-distribution of Squ/DiR@APS NPs (A) and Squ@APS-IR820 NPs (B) after single dose at different time points. *Ex vivo* fluorescence images of Squ/DiR@APS NPs (C) and Squ@APS-IR820 NPs (D) at 8 h and 72 h. Relative tumor targeting index of Squ/DiR@APS and Squ@APS-IR820 at 72 h. Welch's *t*-test was conducted to test for differences between two groups. ∗*p* < 0.05.

Dynamic fluorescence imaging demonstrated that predominant fluorescence accumulated in the liver and spleen during the whole process of observation and reached the maximum at 10 h post dose for Squ/DiR@APS group, with only minimal fluorescence detected in tumor tissue (Fig. 6A).

In contrast, the Squ@APS-IR820 NPs group showed detectable tumor fluorescence as early as 1 h post-injection Fig. 6B. The radiant efficiency gradually increased, reaching its maximum at 48 h (significantly stronger than in other tissues) before slowly declining through 72 h (Fig. 6B).

The good tumor-targeting effects of APS-IR820 modification over APS modification could be more intuitionally manifested by the *ex vivo* fluorescence imaging of the dissected tumors and the major organs. As seen in Fig. 6C and D, in comparison with Squ/DiR@APS group, Squ@APS-IR820 group displayed more biodistribution in tumor while less biodistribution in spleen whatever at the 8 h (n = 1) or the 72 h (n = 3) post dose. The semi-quantitative analysis more clearly showed that the fluorescence intensity ratio (tumor/liver) of was 0.54 for Squ@APS-IR820 NPs, significantly higher than the 0.11 for Squ/DiR@APS NPs (*p* < 0.05, Fig. 6E).

### 3.11 In vivo anti-tumor therapy

*In vitro* anti-tumor effects were evaluated via MTT assay. As shown in Fig. S3A–D, Squ@APS, Squ@APS-IR820, MnO_2_@APS, and MnO_2_@APS-IR820 NPs exhibit significant anti-tumor effects against 4T1 cells. The *in vivo* assay was designed aiming to examine whether Squ-based nanoparticles and MnO_2_-based nanoparticles can exert synergistic anti-tumour effects of chemotherapy and immunotherapy, as well as to testify the effectiveness and safety improvement provided by enhanced tumor targeting due to APS-IR820 modification.

**Fig. 7:**
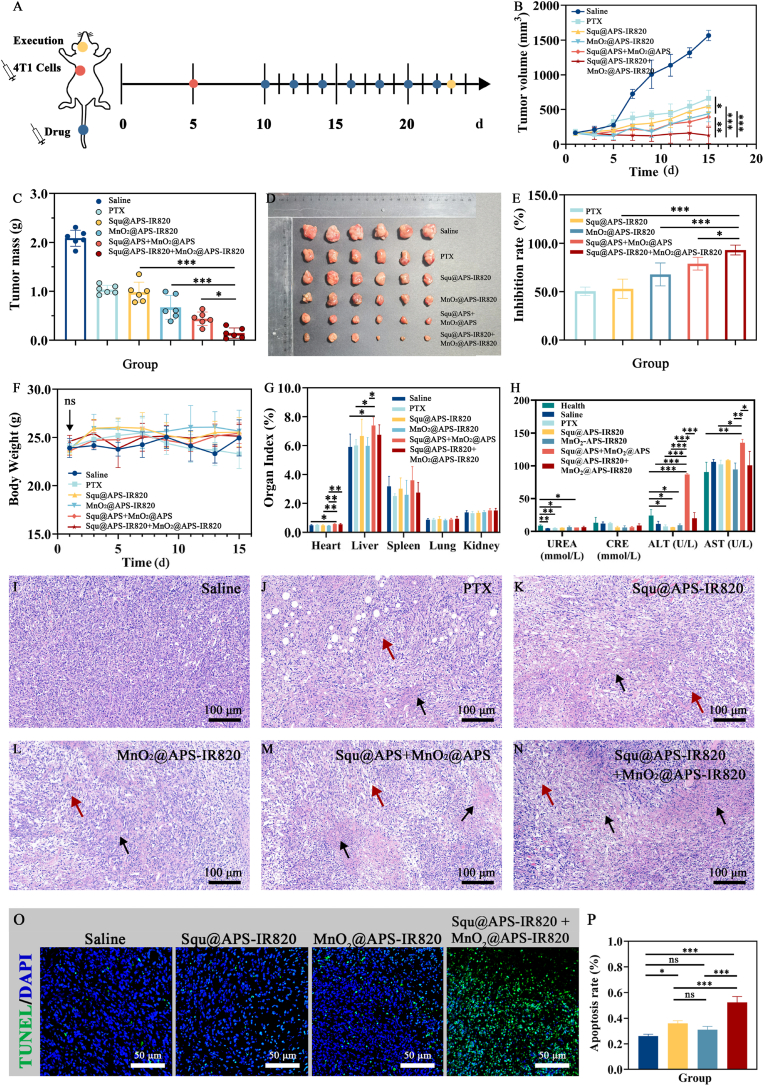
*In vivo* anti-tumor study. (A) Schematic illustration of the work flow. Tumor-growth Curves (B), tumor mass (C), and optical images of tumors (D) of 4T1 tumor bearing mice treated with different formulations. Tumor inhibition rate on basis of tumor weight (E), body weight change (F), and organ index (G) for different groups. ALT, AST, UREA, CRE levels in serum (n = 3, mean ± SD) (H). (I–N) H&E staining of tumor sections. The black arrows point to the necrotic area. The tumor tissue is arranged loosely with enlarged gaps, as indicated by the red arrows. TUNEL staining (O) and apoptosis rate (P) of the tumor tissues in different groups. Differences among groups were calculated by one-way ANOVA analysis. ∗*p* < 0.05, ∗∗*p* < 0.01, and ∗∗∗*p* < 0.001. ns, no significant difference between two groups. (For interpretation of the references to colour in this figure legend, the reader is referred to the Web version of this article.)

When the tumor volume in 4T1 tumor bearing mice reached approximately 150 mm^3^, the mice were randomly divided into 6 groups (n = 6) receiving: normal saline, PTX injections, Squ@APS-IR820 NPs, MnO_2_@APS-IR820 NPs, Squ@ APS NPs + MnO_2_@APS NPs, and Squ@APS-IR820 NPs + MnO_2_@APS-IR820 NPs. Treatments were administered intravenously every other day for a total of seven doses (Fig. 7A). No significant differences in initial body weight were observed between groups (Fig. 7F). Mice in the normal saline group exhibited slow tumor growth during the first 5 days, followed by rapid tumor progression. In contrast, the PTX injection group demonstrated significantly slower tumor growth, with a final mean tumor volume of 658.6 mm^3^ - representing only 57.9 % of the saline control group's volume (Fig. 7B). Tumor growth inhibition was more pronounced in the Squ@APS-IR820 NPs group, followed by the MnO_2_@APS-IR820 NPs group and the Squ@APS NPs + MnO_2_@APS NPs group. Notably, the Squ@APS-IR820 NPs + MnO_2_@APS-IR820 NPs group showed modest tumor regression.

On the next day after the last dose, the mice were anesthetized and executed. The tumors were collected, weighed (Fig. 7C) and photographed (Fig. 7D). Tumor inhibition rates (TIR) based on tumor mass were calculated and analyzed (Fig. 7E). Results demonstrated comparable anti-tumor efficacy between Squ@APS-IR820 NPs and PTX injections. The MnO_2_@APS-IR820 NPs, Squ@APS NPs + MnO_2_@APS NPs, and Squ@APS-IR820 NPs + MnO_2_@APS-IR820 NPs groups all exhibited significantly greater anti-tumor efficacy than the PTX injection group (*p* < 0.001), highlighting the therapeutic potential of MnO_2_-based nanoparticles. The combination of Squ@APS-IR820 NPs and MnO_2_@APS-IR820 NPs showed remarkable enhanced antitumor therapeutic efficacy than Squ@APS-IR820 NPs (*p* < 0.001) or MnO_2_@APS-IR820 NPs (*p* < 0.001), demonstrated synergistic anti-tumor effects between Squ@APS-IR820 NPs and MnO_2_@APS-IR820 NPs. The higher TIR of Squ@APS-IR820 NPs + MnO_2_@APS-IR820 NP group over Squ@APS NPs + MnO_2_@APS NPs group (*p* < 0.05) was probably due to the tumor-targeting effects of APS-IR820.

The tumor tissue of normal saline control group appeared normal, with plenty of tumor structure comprising densely located solid tumor cells without focal necrosis (Fig. 7I). However, necrotic foci were visible in certain areas of the tumor tissue in other treatment groups. The nuclei of necrotic tumor cells were fragmented or disappeared, and the cytoplasm was eosinophilic staining (Fig. 7J–N, black arrows point). Additionally, the surrounding tumor tissue showed notable structural changes, including loose arrangement and increased intercellular spacing, as indicated by red arrows. The greatest therapeutic impact was observed in the Squ@APS-IR820 NPs + MnO_2_@APS-IR820 NPs group (Fig. 7N), in which there was the largest necrotic region with plenty of disintegrated nuclei and the loosening of the tumor tissue corroborated the superior effects. Apoptosis of tumor tissues evaluated via TUNEL staining of tumor sections (Fig. 7O and P) confirmed the synergistically therapeutic effects.

The body weight change is an important indicator of the bio-safety of anti-tumor agents during tumor treatment. As shown in Fig. 7F, all treatment groups exhibited a slight increase in body weight except for the PTX injection group, demonstrating their favorable biosafety profiles.

Organ indices are commonly employed for the evaluation of drug-associated toxicities [44]. As shown in Fig. 7G, there was no significant difference in spleen index, lung index and kidney index among all the groups. However, Squ@APS NPs + MnO_2_@APS NPs group showed a significantly increased heart index than saline, Squ@APS-IR820 NPs, MnO_2_@APS-IR820 NPs group, indicating that the combinational use of Squ@APS NPs and MnO_2_@APS NPs may induce enhanced side effects to hearts. Though there is no significance of heart index between Squ@APS-IR820 + MnO_2_@APS-IR820 NPs group and Squ@APS + MnO_2_@APS NPs group, the mean heart index of Squ@APS-IR820 + MnO_2_@APS-IR820 NPs group is lower. Furthermore, the decreased biodistribution in heart of APS-IR820 coating nanoparticles could be seen in Fig. 6C and D, indicating heart injury could be attenuated by IR820 modification. The liver index also witnessed a significant increase in Squ@APS NPs + MnO_2_@APS NPs group than in normal saline group, PTX injections group and MnO_2_@APS-IR820 NPs group, indicating that Squ@APS NPs + MnO_2_@APS NPs may induce certain damage to liver. However, there is no significance of liver between Squ@APS-IR820 + MnO_2_@APS-IR820 NPs group and other groups, exhibiting a good safety.

Furthermore, safety of preparations was evaluated by detecting ALT, AST, Urea, and Cre in serum. As shown in Fig. 7H, The serum Urea levels of mice in Saline, PTX, Squ@APS-IR820, Squ@APS NPs + MnO_2_@APS NPs groups are significantly lower than in healthy group, while all treatment groups show comparable Urea levels to the saline control group. In addition to the similar Cre levels between healthy and experimental groups, conclusion could be drawn that PTX, Squ@APS-IR820, and Squ@APS NPs + MnO_2_@APS NPs treatments lead to mild kidney damage comparable to saline.

Notably, ALT and AST levels in Squ@APS NPs + MnO_2_@APS NPs group exhibited significantly higher than in other groups, indicating drug-induced hepatotoxicity of Squ@APS NPs + MnO_2_@APS NPs. In contrast, there is no significant difference in ALT and AST levels of mice among Squ@APS-IR820 NPs, MnO_2_@APS-IR820 NPs, Squ@APS-IR820 NPs + MnO_2_@APS-IR820 NPs, and saline groups, demonstrating that IR820 modification effectively mitigates hepatic injury.

These findings collectively demonstrate favorable safety profiles for all treatment regimens except for the unmodified Squ@APS NPs + MnO_2_@APS NPs combination.

### 3.12 Anti-tumor immune responses of the MnO_2_ NPs based chemoimmunotherapy

Our findings reveal that MnO_2_ NPs based chemoimmunotherapy inhibits the growth of the tumor while inducing maturation of DCs and repolarization of macrophages to M1 TAMs as confirmed by the *in vitro* studies. To further investigate the underlying mechanisms, we evaluated the capability of MnO_2_ NPs based therapy to activate immune responses of in tumor bearing mice, taking Squ@APS-IR820 as a control. MnO_2_ is a well investigated potential tumor STING agonist, which could produce Mn^2+^ in response to tumor microenvironment and then activate anti-tumor immunity and relieve the immune suppression. Taking these characteristics into consideration, we hypothesized MnO_2_ NPs based therapy would activating anti-tumor immunity while reducing immune suppression.

**Fig. 8:**
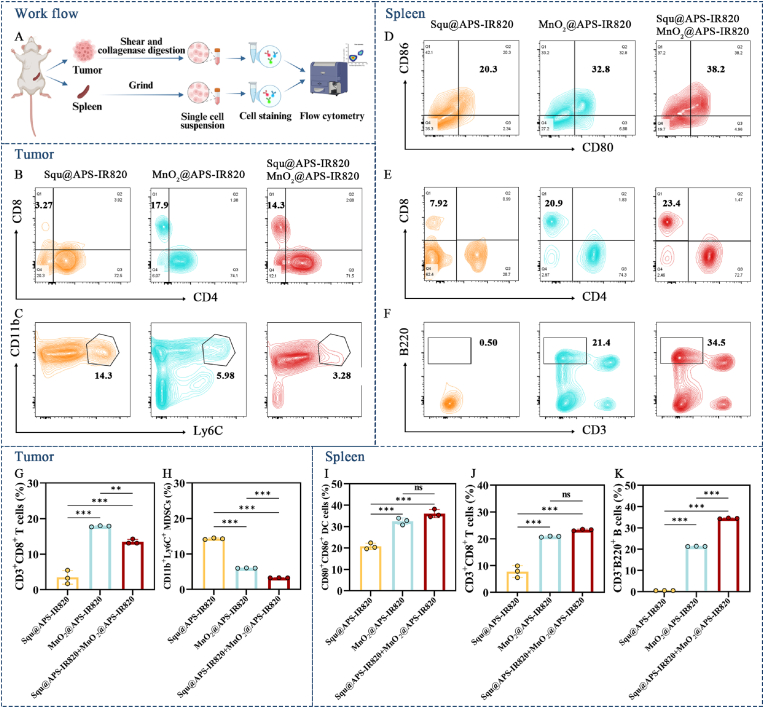
Immunoassay of the tumor and the spleen after different treatments. (A) Workflow of the immunoassay based on Flow Cytometry. Created in BioRender. Gong, T. (2025) https://BioRender.com/tjhnwh2. Representative flow cytometry plots of tumor immune cells including CD3^+^CD8^+^ T cells (B), CD11b^+^ Ly6c^+^ MDSCs (C). Representative flow cytometry plots of splenic immune cells including CD80^+^CD86^+^ DCs (D), CD3^+^CD8^+^ T cells (E), and CD3^-^B220^+^ B cells (F). The quantitative analysis of CD3^+^CD8^+^ T cells (G), CD11b^+^ Ly6c^+^ MDSCs (H) in the tumor and CD80^+^CD86^+^ DCs (I), CD3^+^CD8^+^ T cells (J), and CD3^-^B220^+^ B cells (K) in the spleen after different treatments. Data are presented as mean ± SD. ∗*p* < 0.05, ∗∗*p* < 0.01, and ∗∗∗*p* < 0.001. ns, no significant difference between two groups.

Twenty-four hours following the final dose, the spleen and tumor of mice were collected for the immune cells analysis (Fig. 8A). Spleen was selected to analyze T cells, B cells, and DCs population as the spleen is the largest peripheral immune organ and the residence for mature lymphocyte cells. The gating strategy for flow cytometry analysis of spleen was shown in Fig. S7. The results indicated that the mice treated with MnO_2_@APS-IR820 NPs and Squ@APS-IR820 NPs + MnO_2_@APS-IR820 NPs displayed much more mature DCs (Fig. 8D, I) and CD8^+^ T cell (Fig. 8E, J) in the spleen than the mice treated with Squ@APS-IR820, confirming our hypothesis.

STING agonists activate immune response mainly by enhancing antigen presentation [45]. DCs are the most potent professional APCs, which bridge the innate and adaptive immune [46]. Immature DCs mature in the spleen present antigens to T cells, thus inducing specific antitumor immune responses. Additionally, MnO_2_@APS-IR820 NPs and Squ@APS-IR820 NPs + MnO_2_@APS-IR820 NPs led to a higher percentage of B cells (Fig. 8F, K) in spleen than Squ@APS-IR820 group. Compared with the specific cellular immune response process mediated by T cells, B cells can recognize more types of antigens without the need of antigen processing and presentation by APCs, and are not MHC-restricted, which is an important complement to the specific cellular immune responses [47].

Therefore, MnO_2_-based treatments can activate a potent anti-tumor immune response comparing with Squ@APS-IR820 NPs via activating cGAS-STING pathway. The Western blot results revealed that the expression levels of p-IRF3 and p-TBK1 in MnO_2_@APS-IR820 NPs or Squ@APS-IR820 NPs + MnO_2_@APS-IR820 NPs treated mice were significantly higher compared to those treated with Saline or Squ@APS-IR820 (Fig. S9). These findings suggest that the STING pathway is activated in mice following treatment with MnO_2_@APS-IR820 NPs or Squ@APS-IR820 NPs + MnO_2_@APS-IR820 NPs, with MnO_2_@APS-IR820 NPs playing a dominant role in this activation.

The poor infiltration of immunocytes and the accumulation of immunosuppressive cells in the tumor severely weakened the anti-tumor immune responses [48,49]. Thus, we examined T cells population in tumor and found that MnO_2_@APS-IR820 NPs and Squ@APS-IR820 NPs + MnO_2_@APS-IR820 NPs could significantly enhance the intratumoral T cells infiltration comparing with Squ@APS-IR820 (Fig. 8B, G). The gating strategy for flow cytometry analysis of tumor tissue was shown in Fig. S8. Subsequently, we studied MDSCs present in the tumor tissue in order to assess the efficacy of different treatments in relieving the immunosuppressive tumor microenvironment. As shown in Fig. 8C, H, MnO_2_@APS-IR820 NPs, and Squ@APS-IR820 NPs + MnO_2_@APS-IR820 NPs significantly decreased the population of MDSCs. These MnO_2_ nanoparticle-based treatments demonstrated a higher capability to activate systemic immunity and alleviate immune suppression, thereby offering promising therapeutic strategies for anti-tumor immunotherapy.

In addition, MnO_2_- based NPs offer distinct advantages including simple preparation, high stability, cost-effectiveness, tumor microenvironment responsiveness, potential STING agonist activity, and being administered via intravenous injection after surface modification. These properties overcome the limitations of established STING agonists.

## 4 Conclusion

A simple one-step synthesis method was developed to synthesize MnO_2_ nanoparticles using KMnO_4_ and anhydrous ethanol. IR820, a near-infrared fluorescent probe with intrinsic tumor-seeking capability, was conjugated with APS to synthtize a multifunctional biomaterial. This biomaterial enabled surface modification of MnO_2_ nanoparticles to enhance stability, tumor targeting, and *in vivo* imaging performance while simultaneously encapsulating chemotherapeutic agents such as Squ into tumor-seeking nanoparticles.

*In vitro* study showed that MnO_2_@APS NPs were able to generate Mn^2+^ in response to H^+^ and GSH, which were elevated in tumor microenvironment. Furthermore, MnO_2_@APS-IR820 nanoparticles exhibited remarkable tumor microenvironment responsiveness, with the released Mn^2+^ promoting DCs maturation and successfully reprogramming tumor-associated macrophages (TAMs) from an M2 immunosuppressive phenotype to an M1 anti-tumor phenotype. Therefore, the combination treatment of Squ@APS-IR820 and MnO_2_@APS-IR820 achieved synergistic therapeutic efficacy in the 4T1 tumor mouse model, with a tumor inhibition rate exceeding 90 %. Flow cytometry analysis revealed that MnO_2_-based treatments effectively activated systematic immune and improved tumor microenvironment. In addition, analyzed by flow cytometry, MnO_2_-based treatments activated immune cells at the spleen and tumor site. The approach significantly reduced immunosuppressive myeloid-derived suppressor cells (MDSCs) while substantially increasing anti-tumor immune cells, including m DCs, CD8^+^ T cells, and B cells, turning the “cold” tumor microenvironment “hot”. Furthermore, Squ@APS-IR820 + MnO_2_@APS-IR820 does not appear significant toxic side effects.

In conclusion, our findings indicate that Squ@APS-IR820 + MnO_2_@APS-IR820 strategy have great potential for potent tumor immunotherapy. Besides, MnO_2_ NPs have several advantages: simple synthesis, environmental friendliness, low cost, readily available raw materials, easy surface modification, and multifunctionality. They can be used as an open basic research material with extended function after surface modification, or synergistically combined with other therapies for tumor therapy, and have enormous research value.

## CRediT authorship contribution statement

**Tingting Gong:** Writing – review & editing, Writing – original draft, Visualization, Methodology, Investigation, Formal analysis, Data curation, Conceptualization. **Xiaohuan Wang:** Writing – review & editing, Methodology, Investigation. **Ziqi Liu:** Investigation, Data curation. **Pengxin Li:** Visualization, Software. **Yunqian Lu:** Investigation, Data curation. **Yaoyao Guo:** Visualization, Data curation. **Meihua Han:** Writing – review & editing, Resources, Project administration, Funding acquisition. **Xiangtao Wang:** Writing – review & editing, Validation, Supervision, Resources, Project administration, Funding acquisition, Conceptualization.

## Declaration of competing interest

The authors declare that they have no known competing financial interests or personal relationships that could have appeared to influence the work reported in this paper.

## Data Availability

Data will be made available on request.

## Abbreviations

TME: Tumor microenvironment
APS: Astragalus polysaccharides
Squ: Squamocin
DCs: Dendritic cells
TAMs: Tumor accociated macrophages
CTL: Cytotoxic lymphocytes
OA: Oleic acid
RTTI: Relative tumor targeting index
APCs: Antigen presenting cells
PDI: Polydispersity index
MTT: 3-(4,5-dimethylthiazol-2-yl)-2,5-diphenyltetrazolium bromide

## Appendix A Supplementary data

Optimization of reaction conditions of preparing MnO_2_ NPs;Chemical structure of Squamocin.

Particle size and PDI changes of of MnO_2_ NPs made of Ethanol versus Methanol;

The cytotoxicity of Squ@APS, Squ@APS-IR820, MnO_2_@APS, MnO_2_@APS-IR820 nanoparticles against 4T1 cells.

The roles of OATP to Squ/C6@APS NPs uptake.

The gating strategy for flow cytometry analysis of BMDC & BMDM.

Biodistribution of IR820 in 4T1 tumor-bearing mice.

The gating strategy for flow cytometry analysis of spleen.

The gating strategy for flow cytometry analysis of tumor tissue.

Activation of the cGAS-STING pathway.
