# Supplementary material for: A MnO2-based tumor-seeking nanoplatform for enhanced chemoimmunotherapy against 4T1 breast cancer
Source: Mater Today Bio. 2025 Jun 17;33:102000. doi: 10.1016/j.mtbio.2025.102000 (PMC12272469; doi:10.1016/j.mtbio.2025.102000)
Supplement: Multimedia component 1 [file mmc1.docx]

**Supplementary material**

**A MnO_2_-based tumor-seeking nanoplatform for enhanced chemoimmunotherapy against 4T1 breast cancer**

Tingting Gong^a^, Xiaohuan Wang^b^, Ziqi Liu^a^, Pengxin Li^c^, Yunqian Lu^a^, Yaoyao Guo^a^, Meihua Han ^a*^, Xiangtao Wang^a*^

**Reagents**

Anti-IRF-3 antibody, anti-TBK-1 antibody, Recombinant Anti-GAPDH antibody (Rabbit mAb), HRP conjugated Goat Anti-Rat IgG (H+L) were purchased from ServiceBio (Hubei, China). Anti-phospho-IRF-3 antibody was obtained from Affinity Biosciences (Jiangsu, China). Anti-phospho-TBK-1 antibody was provided by Cell Signaling Technology (MA, United States). RIPA lysis buffer, Phenylmethylsulfonyl fluoride, Phosphatase Inhibitor Cocktail, BCA Protein Assay Kit, 5× SDS-PAGE Loading Buffer, Prestained Protein Marker Ⅶ (8-195 kDa), PVDF Membrane (0.45 μm), Protein-Free Blocking Buffer, ECL Western Blotting Substrate were purchased from ServiceBio (Hubei, China).

**Cell Cytotoxicity Assay**

The cytotoxicity of Squ@APS, Squ@APS-IR820, MnO_2_@APS, MnO_2_@APS-IR820 NPs was evaluated by MTT assay on 4T1 cell line. Cells were cultivated to logarithmic growth stage and inoculated at a density of 4000 cells per well into a 96 well plate, culturing for 24 h. Gradient diluent of nanoparticles was added to the plate, with 3 replicates per concentration and 200 μl per well; Cells were cultured for another 72 h. Then 10 μl MTT (5mg/ml) was add to each well, continue to culture in the incubator for 3 hours. Culture medium was removed and 100 μl DMSO was added to each well. The absorbance value was measured at 490 nm after being shaken to dissolve thoroughly, IC50 (half maximal inhibitory concentration) of Squ and NPs was calculated.

**Biodistribution of IR820**

Breast cancer mouse model was established by subcutaneously injecting 1.0×10^6^ 4T1 cells into the right axillary region of female Balb/c mice. Three tumor-bearing mice were intravenously administrated with IR820 (1 mg/kg), and fluorescently imaged using the *in vivo* Imaging System (PerkinElmer) at 1, 2, 4, 8, 10, 24, 48, and 72 h post dose (λ_ex_ = 745 nm, λ_em_ = 820 nm). The mice were euthanized at the 72-h mark to collect the tumors, hearts, livers, spleens, lungs, and kidneys for fluorescent imaging applying the IVIS Living Image system as described above. Average radiant efficiency was analyzed using Image analysis software of IVIS Living Image system.

**Western Blot Analysis**

Subcutaneous tumors of the mice were isolated and tumor cells were extracted for western blotting (WB) assay to examine the expression of the cGAS-STING pathway-related proteins: IRF-3, pIRF-3, TBK1, pTBK1. Tumor cells were lysed in RIPA lysis buffer containing protease inhibitors, with total protein quantified using a BCA assay. Loading buffer was added to the mixture, then the mixture was boiled to denature the protein. The denatured protein was then separated by sodium dodecyl sulfate-polyacrylamide gel electrophoresis (SDS-PAGE) and transferred to a polyvinylidene difluoride (PVDF) membrane. Membranes were blocked with protein-free blocking buffer, then incubated overnight at 4°C with primary antibodies (1:1,000). Wash off unbound antibodies with TBST. Then the membrane was incubated with HRP-conjugated secondary antibodies (1:3,000) at room temperature (20-25°C) for 30 min. After washing off unbound antibodies with TBST, blot bands were visualized using chemiluminescent substrate and imaged using Chemiluminescence Imaging System (Servicebio, China), with all experiments including biological replicates (n = 3).

**Table. S1** **Optimization of reaction conditions of preparing MnO_2_ NPs.**

| No | Reductant | C_KMnO4_(mg/ml) | DI water: alcohol (V:V) | Stirring speed (rpm) | Temperature (℃) | Size (nm) | PDI |
| --- | --- | --- | --- | --- | --- | --- | --- |
| 1 | methanol | 1 | 1:1 | 200 | 26 | 191.5±3.905 | 0.189 |
| 2 | ethanol | 1 | 1:1 | 200 | 26 | 272.3±5.537 | 0.065 |
| 3 | ethanol | 1 | 1:1 | 200 | 26 | 149.7±3.342 | 0.213 |
| 4 | ethanol | 2 | 1:1 | 200 | 26 | 166.4±0.987 | 0.255 |
| 5 | ethanol | 3 | 1:1 | 200 | 26 | 156.5±5.279 | 0.169 |
| 6 | ethanol | 4 | 1:1 | 200 | 26 | 174.7±2.902 | 0.145 |
| 7 | ethanol | 5 | 1:1 | 200 | 26 | 220.9±4.661 | 0.173 |
| 8 | ethanol | 3 | 1:1 | 100 | 26 | 150.7±3.989 | 0.204 |
| 9 | ethanol | 3 | 1:1 | 200 | 26 | 175.0±4.636 | 0.237 |
| 10 | ethanol | 3 | 1:1 | 300 | 26 | 161.7±2.443 | 0.199 |
| 11 | ethanol | 3 | 1:1 | 400 | 26 | 170.1±1.457 | 0.163 |
| 12 | ethanol | 3 | 1:1 | 500 | 26 | 189.4±1.589 | 0.228 |
| 13 | ethanol | 3 | 1:1 | 400 | 26 | 161.8±2.318 | 0.246 |
| 14 | ethanol | 3 | 1:2 | 400 | 26 | 167.1±1.582 | 0.195 |
| 14 | ethanol | 3 | 1:3 | 400 | 26 | 172.5±1.793 | 0.134 |
| 14 | ethanol | 3 | 2:1 | 400 | 26 | 245.4±7.615 | 0.493 |
| 14 | ethanol | 3 | 3:1 | 400 | 26 | 272.7±13.87 | 0.438 |
| 14 | ethanol | 3 | 1:3 | 400 | 26 | 150.2±2.193 | 0.188 |
| 14 | ethanol | 3 | 1:3 | 400 | 37 | 158.2±2.458 | 0.187 |
| 14 | ethanol | 3 | 1:3 | 400 | 50 | 210.5±3.980 | 0.217 |


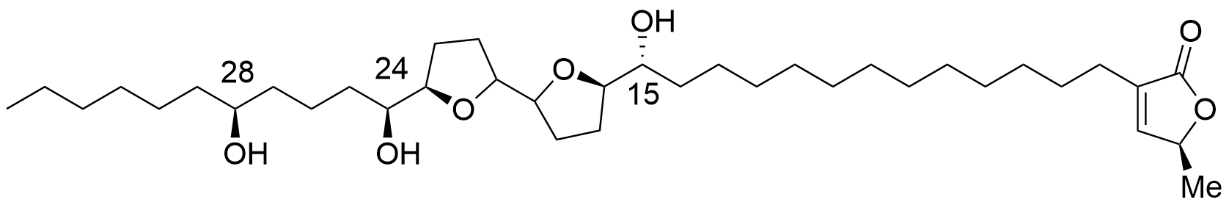


Fig. S1. Chemical structure of Squamocin.


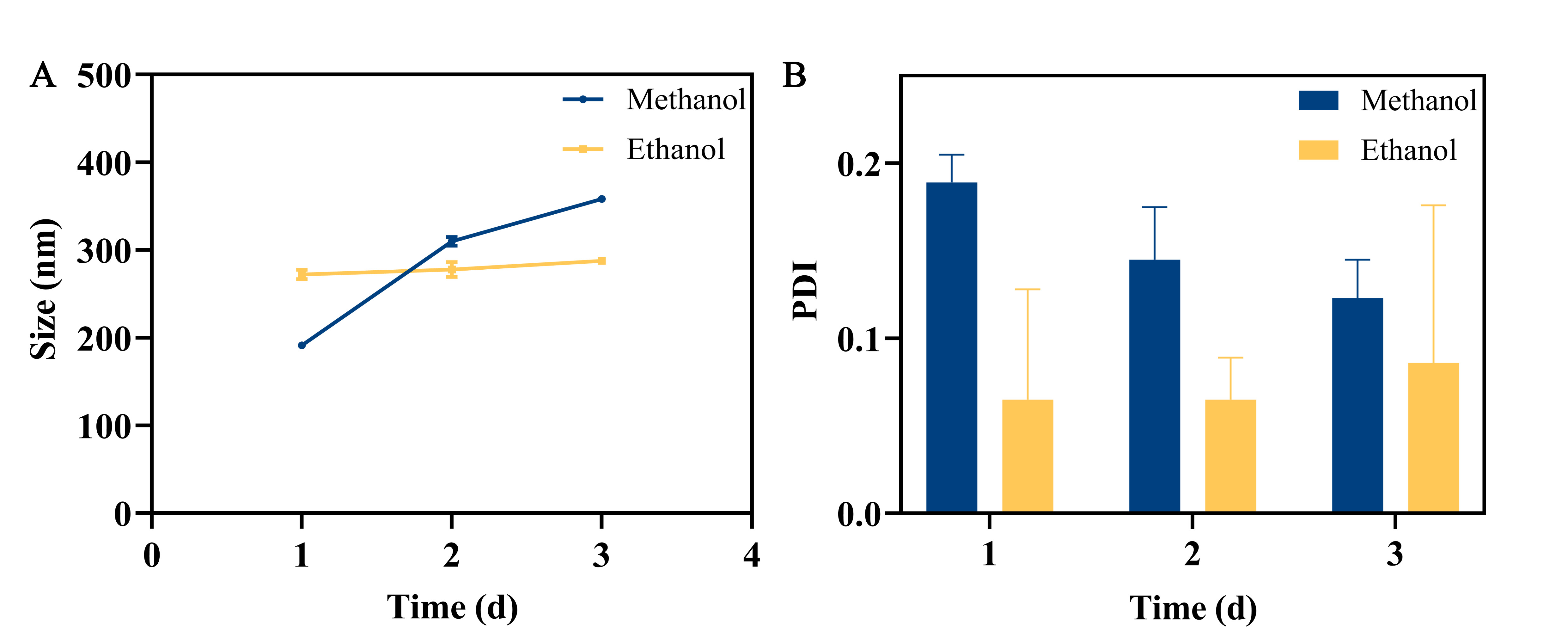


Fig. S2. Particle size and PDI changes of of MnO_2_ NPs made of Ethanol versus Methanol.


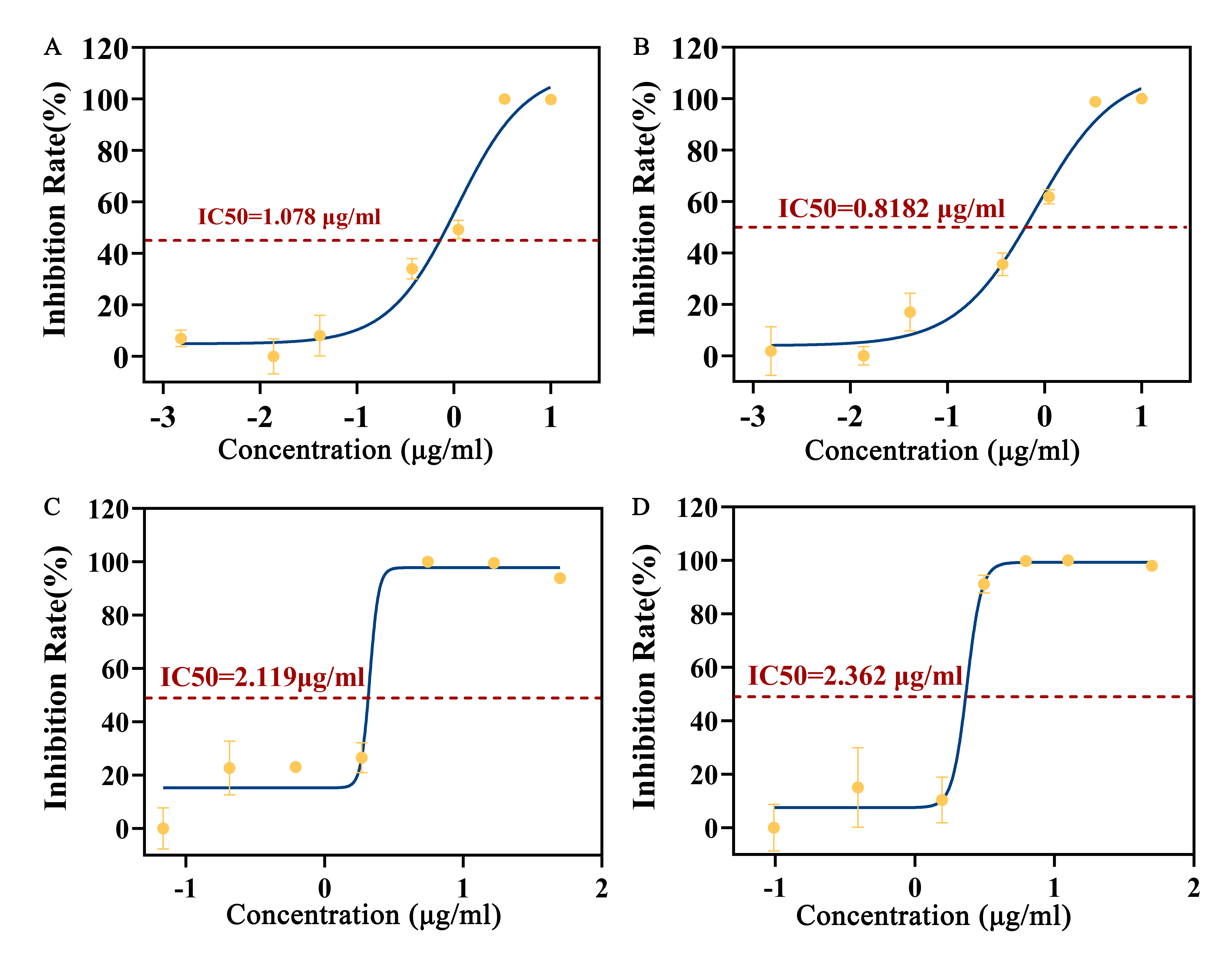


Fig. S3. Inhibition rate of 4T1 cells co-cultured with different NPs: (A) Squ@APS NPs. (B) Squ@APS-IR820 NPs. (C) MnO_2_@APS NPs. (D) MnO_2_@APS-IR820 NPs.

Fig. S4. The roles of OATP to Squ/C6@APS NPs uptake. Fluorescence image (A) and the mean fluorescence intensity (MFI) value (B) of 4T1 cells co-cultured with Squ/C6@APS NPs for 3 h pretreated with 100 μM of probenecid and 50 μM of doxorubicin for 30 min.

**
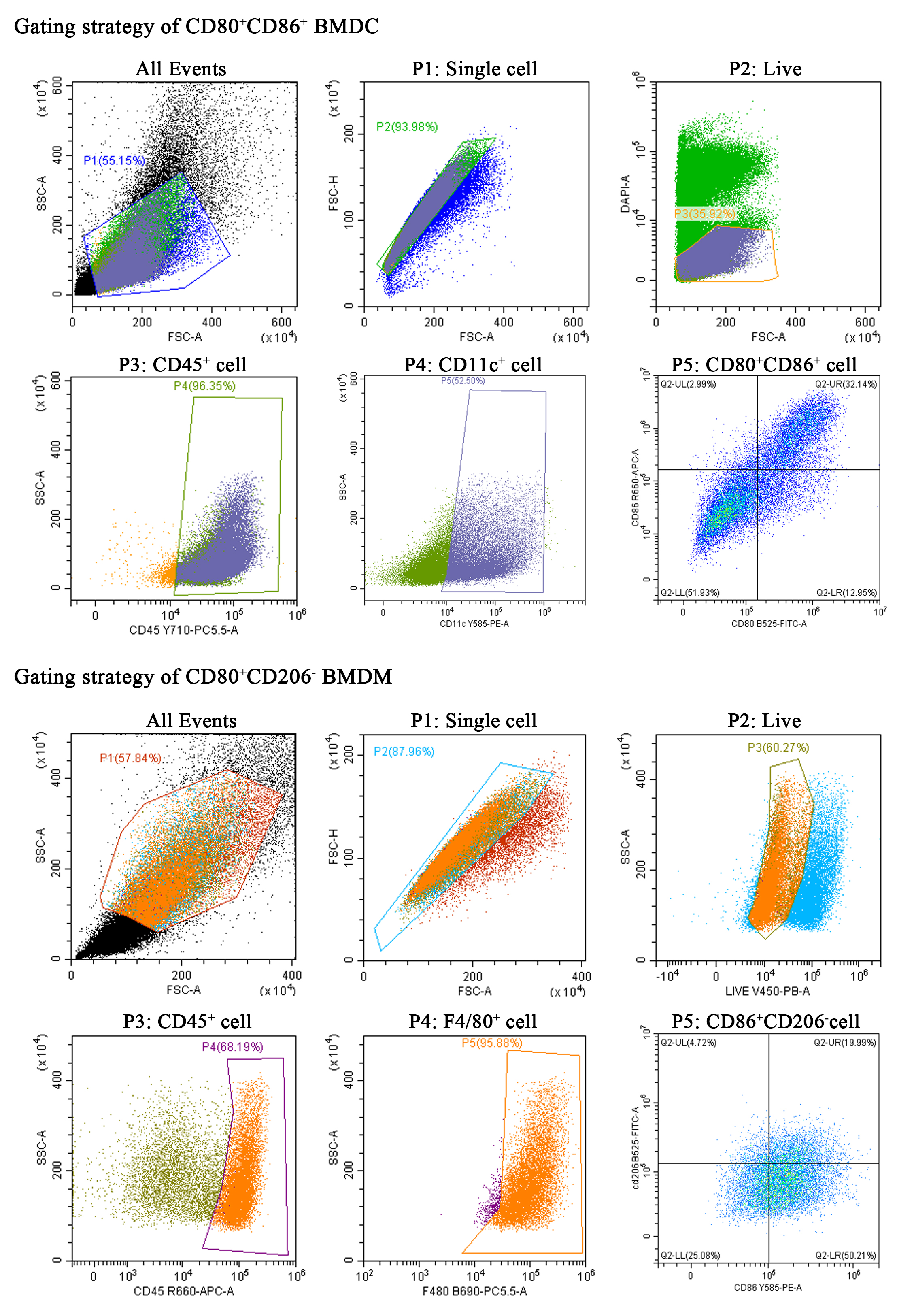
**

Fig. S5. The gating strategy for flow cytometry analysis of BMDC & BMDM.

Fig.S6. Biodistribution of IR820 in 4T1 tumor-bearing mice. Fluorescence images of dynamic bio-distribution of IR820 (A) after single dose at different time points. *Ex vivo* fluorescence images of IR820 (B) at 72 h. Average radiant efficiency of tumor and liver at 72 h (C). Welch’s t test was conducted to test for differences between tumor and liver. ****p* < 0.001.

**
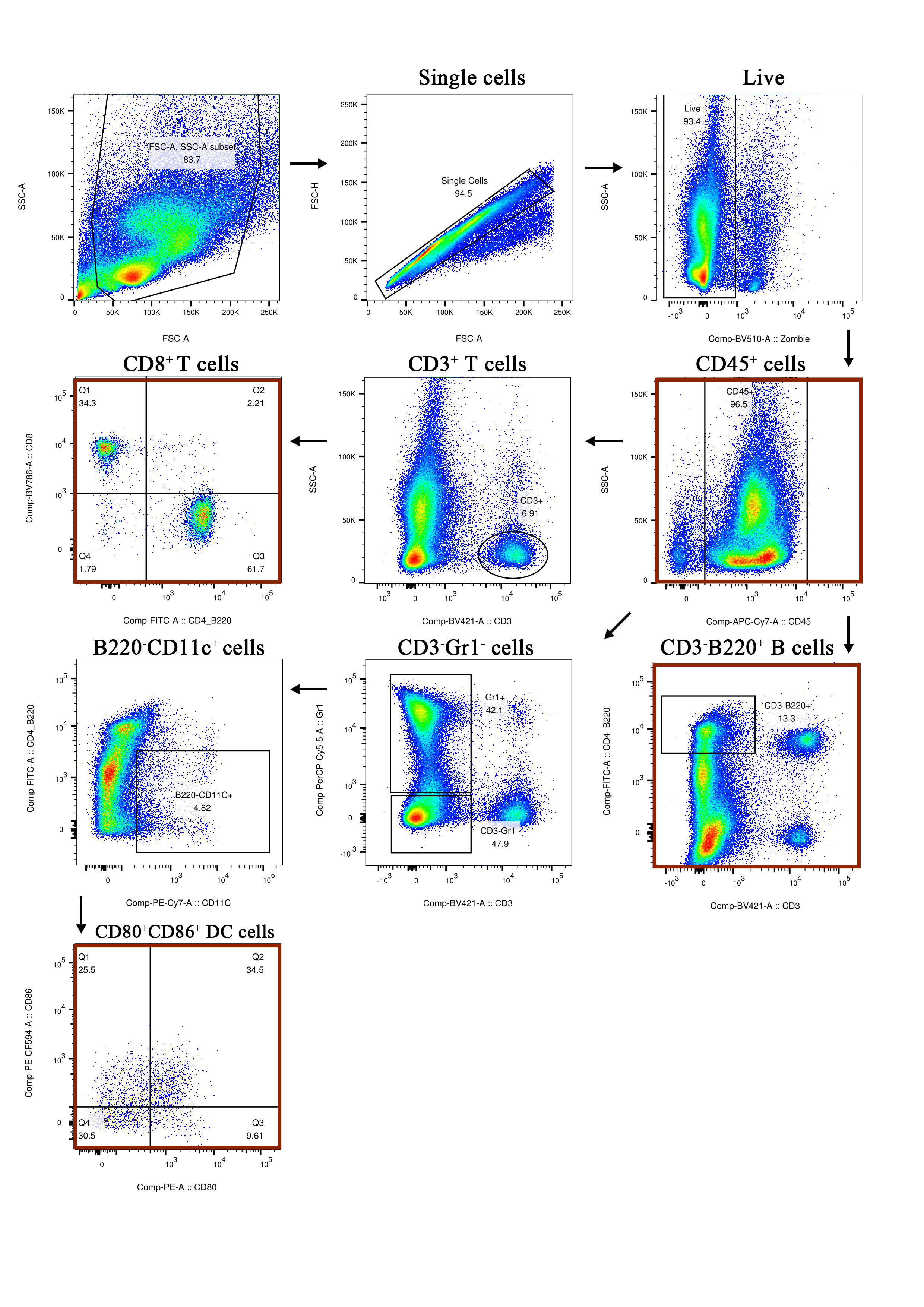
**Fig. S7. The gating strategy for flow cytometry analysis of spleen.

**
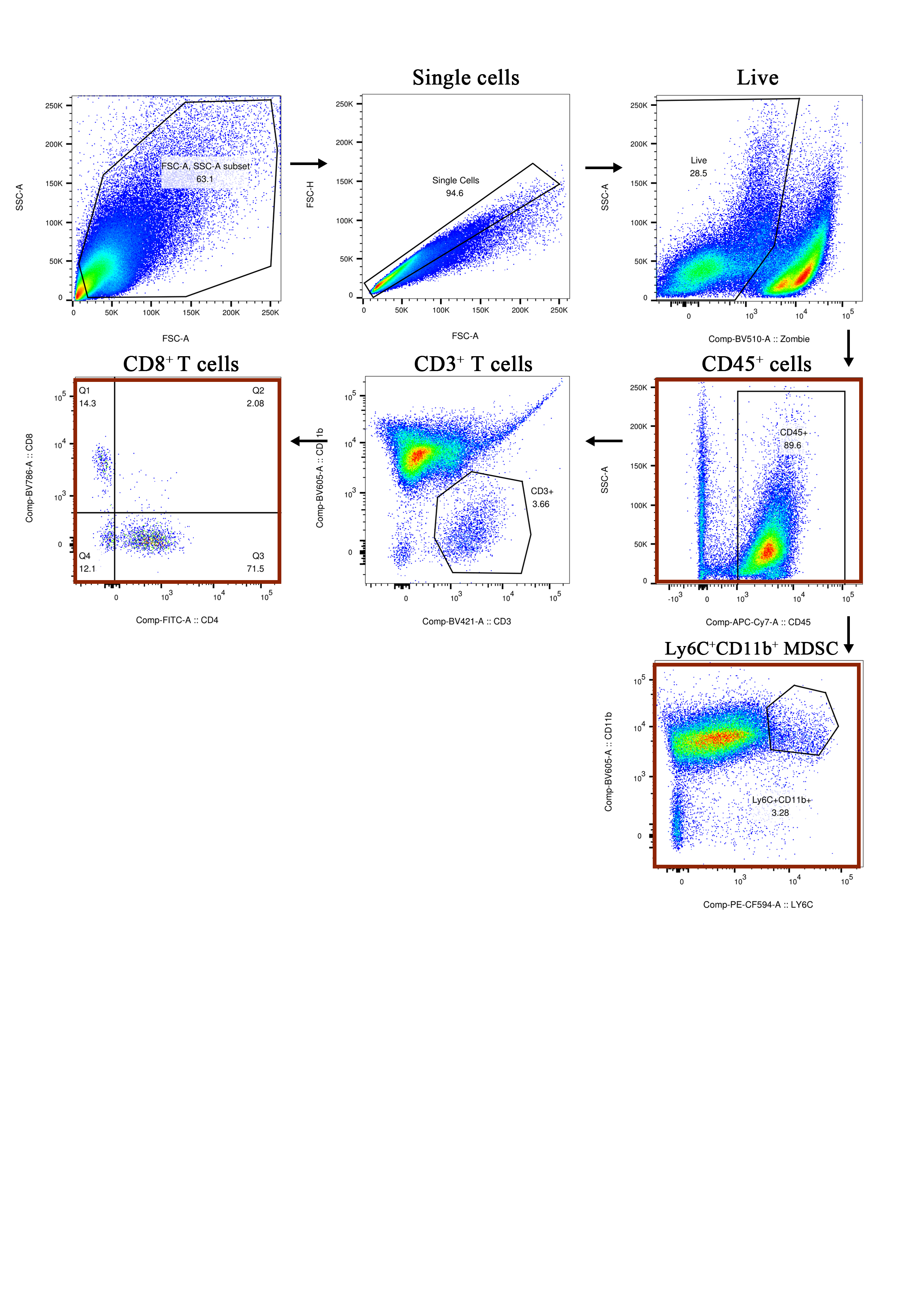
**

Fig. S8. The gating strategy for flow cytometry analysis of tumor tissue.


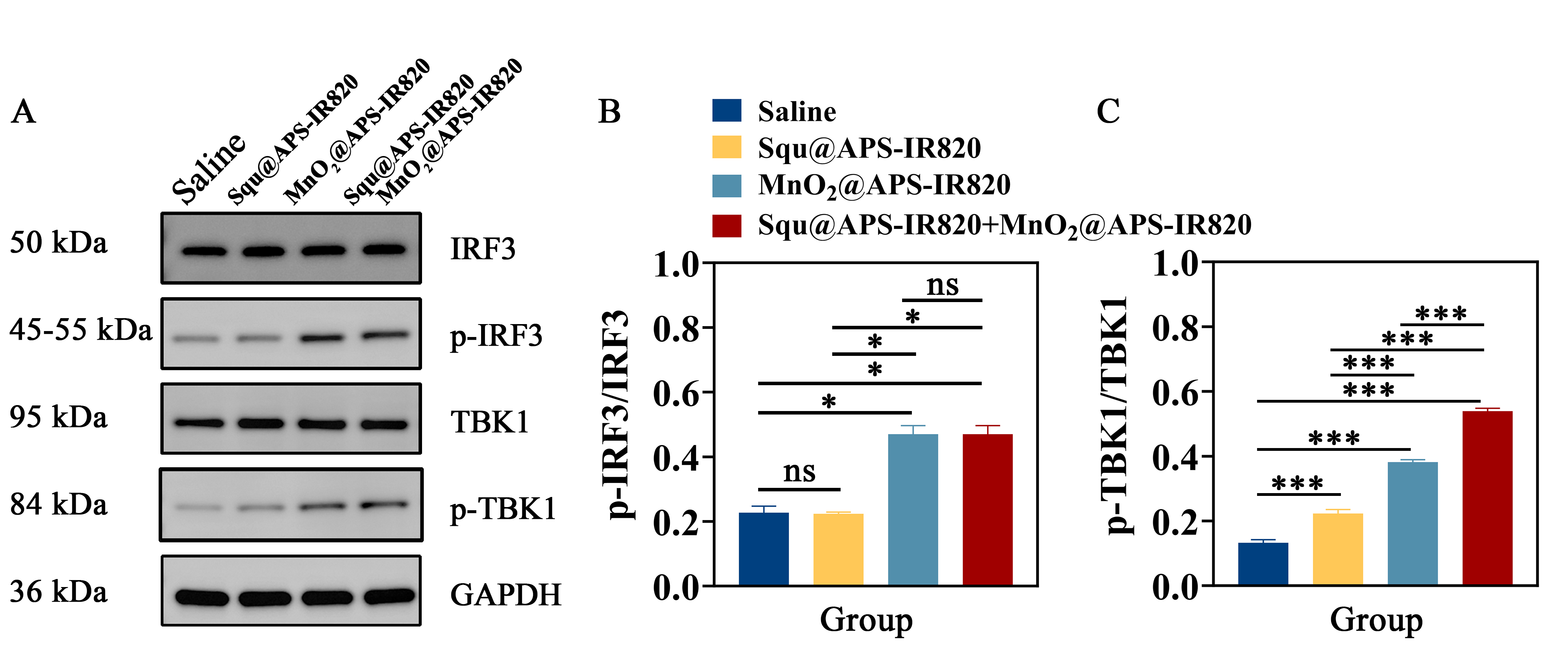


Fig.S9. Activation of the cGAS-STING pathway. (A) Western blot analysis of the expressions of key proteins of the STING pathway in ex vivo tumor tissues including IRF3, p-IRF3, TBK1, p-TBK1 after administration with Saline, Squ@APS-IR820, MnO_2_@APS-IR820, and Squ@APS-IR820 + MnO_2_@APS-IR820. GAPDH was used as the loading control. Relative protein expression of Western blot analysis: (B) p-IRF3/IRF3 (n = 3), Kruskal-Wallis test. (C) p-TBK1/TBK1 (n = 3), One-way ANOVA analysis. *P < 0.05; **P < 0.01; ***P < 0.001; and ns, no difference between two groups.
